# Network pharmacological mechanisms of *Vernonia anthelmintica (L.)* in the treatment of vitiligo: Isorhamnetin induction of melanogenesis via up-regulation of melanin-biosynthetic genes

**DOI:** 10.1186/s12918-017-0486-1

**Published:** 2017-11-16

**Authors:** Ji Ye Wang, Hong Chen, Yin Yin Wang, Xiao Qin Wang, Han Ying Chen, Mei Zhang, Yun Tang, Bo Zhang

**Affiliations:** 1 Pharmacology department, School of Pharmacy, Shihezi University, Shihezi, 832002 China; 2 Key Laboratory of Xinjiang Phytomedicine Resource and Utilization, Ministry of Education, Shihezi University, Shihezi, 832002 China; 3 Shanghai Key Laboratory of New Drug Design, School of Pharmacy, East China University of Science and Technology, 130 Meilong Road, Shanghai, 200237 China

**Keywords:** AdmetSAR, Isorhamnetin, Kaempferide, Melanogenesis, Substructure-drug-target network-based inference, *Vernonia anthelmintica (L.)*, Vitiligo

## Abstract

**Background:**

Vitiligo is a long-term skin disease characterized by the loss of pigment in the skin. The current therapeutic approaches are limited. Although the anti-vitiligo mechanisms of *Vernonia anthelmintica (L.)* remain ambiguous, the herb has been broadly used in Uyghur hospitals to treat vitiligo. The overall objective of the present study aims to identify the potential lead compounds from *Vernonia anthelmintica (L.)* in the treatment of vitiligo via an oral route as well as the melanogenic mechanisms in the systematic approaches in silico of admetSAR and substructure-drug-target network-based inference (SDTNBI).

**Results:**

The results showed that the top 5 active compounds with a relatively higher bioavailability that interacted with 23 therapeutic targets were identified in *Vernonia anthelmintica (L.)* using admetSAR and SDTNBI methods. Among these compounds, Isorhamnetin and Kaempferide, which are methyl-flavonoids, performed 1st and 2nd. Isorhamnetin and Kaempferide significantly increased the expression of melanin-biosynthetic genes (MC1R, MITF, TYR, TYRP1 and DCT) and the tyrosinase activity in B16F10 cells. Isorhamnetin and Kaempferide significantly increased the mRNA-expression of melanin-biosynthetic genes (MC1R, MITF, TYR, TYRP1 and DCT), the protein level of MITF and the tyrosinase activity. Based on the SDTNBI method and experimental verification, Isorhamnetin and Kaempferide effectively increased melanogenesis by targeting the MC1R-MITF signaling pathway, MAPK signaling pathway, PPAR signaling pathway (PPARA, PPARD, PPARG), arachidonic acid metabolism pathway (ALOX12, ALOX15, CBR1) and serotonergic synapses (ALOX12, ALOX15) in the treatment of vitiligo from a network perspective.

**Conclusion:**

We identified the melanogenic activity of the methyl-flavonoids Isorhamnetin and Kaempferide, which were successfully predicted in a network pharmacological analysis of *Vernonia anthelmintica (L.)* by admetSAR and SDTNBI methods.

**Electronic supplementary material:**

The online version of this article (10.1186/s12918-017-0486-1) contains supplementary material, which is available to authorized users.

## Background

Vitiligo is an acquired, progressive depigmentation disorder characterized by the appearance of circumscribed white macules on the skin [1]. Approximately 1% of individuals are affected by vitiligo worldwide [2]. Some populations have rates as high as 2–3% [3]. Many theories, such as autoimmunity, neural and genetic theories, impaired melanocyte migration and/or proliferation, and oxidative stress, have been proposed to explain the mechanism of pigmentation loss [4].

Currently, the main purpose of clinical treatment is to increase the melanogenesis of melanocytes in skin lesions and restore skin color to the normal level [5]. Several treatment options aim to restore pigmentation, including excimer laser, vitamin D analogs and steroid therapy [6]. Unfortunately, these treatments are not widely employed because they induce long-term side effects [7]. Thus, increasing research has focused on the identification of novel therapeutic medicine and the assessment of multi-targets to explain the complex network of the therapeutic mechanism of vitiligo.

Traditional Uyghur Medicine (TUM), particularly *Vernonia anthelmintica (L.)*, which only grows in the high altitude localities in the northwest of China, has been successfully used in the treatment of vitiligo in China [8]. According to the TUM theory, *Vernonia anthelmintica (L.)*, a traditional herb medicine, can treat vitiligo by eliminating abnormal Balgham Hilit (damp and cold) in Uyghur hospitals [9]. However, as a result of the complexity of multi-ingredients and the lack of systematic approaches, its mechanisms remain unclear.

Recently, systematic approaches in silico based on network pharmacology have made a significant contribution to disclose the multi-targets of modern medicine through pharmacokinetic evaluation, target prediction and network/pathway analysis [10]. The admetSAR and substructure-drug-target network-based inference (SDTNBI) methods have been validated as successful for the study of Western medicine, particularly in the new use of old drugs and the development of new indications [11, 12]. However, these powerful tools are rarely employed in TUM.

The systematic in silico approaches of admetSAR and SDTNBI were employed in the present study. Key molecules were further identified via in vitro methods. The network pharmacological mechanism of *Vernonia anthelmintica (L.)* will provide a novel understanding for vitiligo treatments.

## Methods

**Fig. 1:**
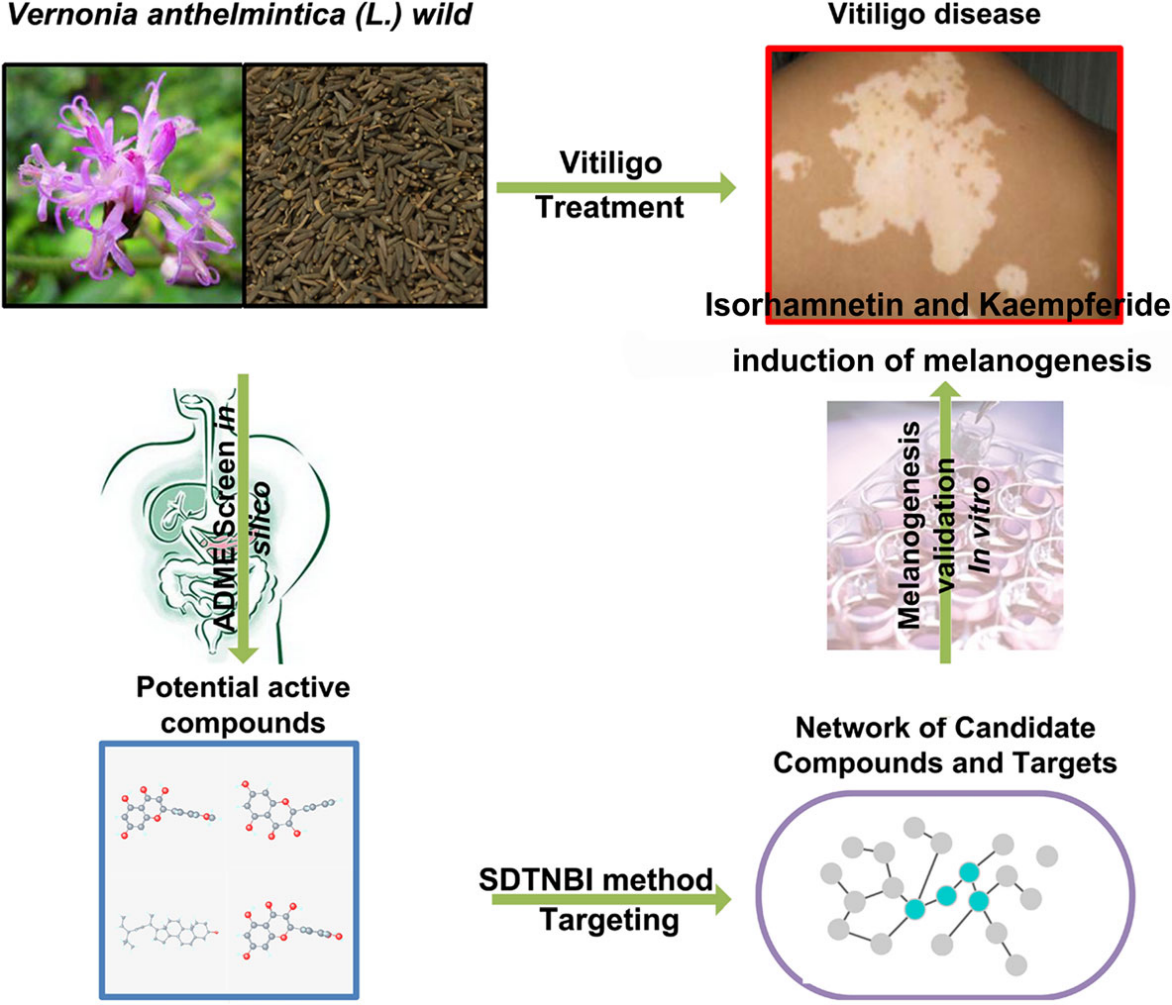
Diagram of the pathway-based SDTNBI and admetSAR approaches developed to identify the network pharmacological mechanisms of *Vernonia anthelmintica (L.)* for the treatment of vitiligo

The entire pathway-based on SDTNBI and admetSAR methods is illustrated in Fig. 1.

### Chemical library construction

Based on a review of *Vernonia anthelmintica (L.)*, all chemicals of *Vernonia anthelmintica (L.)* were collected from the TCMID database (http://www.megabionet.org/tcmid), the Chinese academy of sciences Chemistry Database (http://www.organchem.csdb.cn) and the TCMSP database (http://ibts.hkbu.edu.hk/LSP/tcmsp.php). All of the compounds collected were normalized to the canonical SMILES format.

### Potentially active compounds sorting via admetSAR method

TUM has been successfully used via the oral route in the treatment of vitiligo in China. Among the pharmacokinetic properties of ADME, the absorption and metabolism were the most important factors that affected oral bioavailability [13]. A better therapeutic drug or lead compound should not only have positive parameters of human intestinal absorption (HIA) and Caco-2 permeability (Caco-2) in absorption but also inhibitory parameters of the five major CYP isoforms (CYP450 1A2, CYP450 2C9, CYP450 2C19, CYP450 2D6 and CYP450 3A4) in metabolism. We used an open source, comprehensive computer readable database, namely, admetSAR, to filter or predict the absorption and metabolism-associated properties of all molecules (http://lmmd.ecust.edu.cn/admetsar1/) [13]. The metabolism of drugs and the calibration of the inhibitory parameters of the five major CYP isoforms were comprehensively calculated with the following equation [14].

1 $$ score={\sum}_k(result)(Q) $$

Equation (1) sums the results and overall predictive accuracy (*Q*) of each compound, with five major CYP isoforms (index *k*). The overall predictive accuracies were as follows: CYP450 1A2 Inhibitor (*Q* = 0.8147), CYP450 2C9 Inhibitor (*Q* = 0.8018), CYP450 2D6 Inhibitor (*Q* = 0.8551), CYP450 2C19 Inhibitor (*Q* = 0.8054) and CYP450 3A4 Inhibitor (*Q* = 0.6450).

### Building the compound-target interaction via the SDTNBI method

The targets of the potentially active compounds were predicted using the SDTNBI method, an integrated network and chemoinformatic tool for the systematic prediction of compound-target interactions, particularly new chemical entities [11]. For this purpose, the canonical SMILES format was converted into the substructure fingerprint (FP4) format using PaDEL-Descriptor software (version 2.18) [15]. For each compound, the top 20 predicted targets were stored as putative targets. The targets were subsequently normalized to the official gene name using the UniProt database (http://www.uniprot.org/) as previously described.

The detailed command lines for prediction, cross validation and external validation are presented on our website (http://lmmd.ecust.edu.cn/methods/sdtSDTNBI/).

### Identification of keratinocyte- and melanocyte-specific targets

To analyze the target distribution within the keratinocytes and melanocytes, we examined the protein expression data from the Human Protein Atlas database (http://www.proteinatlas.org/), a database of tissue microarray images labeled with antibodies against 11,250 human proteins [16]. We selected the human species and sorted the target expression based on their protein expression levels in keratinocytes and melanocytes.

### Network construction and bioinformatics analysis

For the sake of interpreting the therapeutic mechanisms of *Vernonia anthelmintica (L.)* for vitiligo at a network level, we constructed the compound-target interaction (CTI) network. All active compounds in *Vernonia anthelmintica (L.)* and their screening targets were utilized to generate a bipartite graph of CTIs, in which a compound and a target are linked with each other if the compound targets the protein or relevant regulators. The bioinformatic functional interaction network analysis of the keratinocyte- and melanocyte-specific targets was determined using the DAVID database (https://david.ncifcrf.gov/home.jsp) [17]. The Protein-Protein interaction network was identified by the STRING database (http://string-db.org/) [18]. All visualized network graphs were constructed by Cytoscape v3.4.0, an open software package project used to visualize, integrate, model, and analyze molecular and genetic interaction networks [19].

### Cell culture

The B16F10 melanoma cell line was purchased from the Cancer Cell Repository (Shanghai Cell Bank, China). Cells were cultured in DMEM containing 10% fetal bovine serum and 1% penicillin/streptomycin in a 5% CO_2_ humidified incubator at 37 °C (Gibco BRL, USA).

### Cell viability assay

The general viability of the cultured cells was determined through the reduction of 3-(4,5-dimethyl-thiazol-2-yl)-2,5-diphenyl tetrazolium bromide (MTT) to formazan [20]. Exponentially growing B16F10 cells were trypsinized, harvested and equal numbers of cells (2 × 10^5^ cells/ml) in 100 μl medium were plated in 96-well microplates. After overnight incubation, 100 μl of different concentrations (8, 16, 32 μM) of Isorhamnetin and Kaempferide (purity >98%; Shanghai yuanye Bio-Technology Co., Ltd., China) was added to the well for 24 h. The untreated controls were exposed to fresh medium. Following this, 50 μl 5 mg/ml MTT solution was added to each well and incubated for 4 h. After aspirating the culture medium, the resulting formazan was dissolved with 150 μl dimethylsulfoxide (Sigma, USA). The plates were then placed on a shaker for 5 min and measured at 570 nm by using a microplate reader (Thermo Varioskan Flash 3001, USA).

### Measurement of melanin contents

The melanin contents were measured in extracellular and intracellular according to a previously described method [21]. The B16F10 cells were treated with 100 μM 8-MOP (purity >98%; Shanghai yuanye Bio-Technology Co., Ltd., China) and Isorhamnetin and Kaempferide (8, 16, 32 μM) for 24 h. The cells and the supernatant were collected respectively. In extracellular assay, one milliliter each of 400 μM 2-[4-(2-hydroxyethyl) piperazin-1-yl] ethanesulfonic acid buffer (pH 6.8) and EtOH (9:1, *v*/v) was added to 1 mL medium. The optical density (OD) was measured at 405 nm after a calibration curve obtained from a synthetic melanin solution. The cells were collected pelleted and washed twice with PBS (Shanghai Sangong Co., China). Cells were collected by centrifugation at 5000 rpm for 10 min. After digestion in 1 mL of 1 N NaOH solution for 1 h at 80 °C, the intracellular melanin was measured as described above.

### Cellular tyrosinase activity assay

Tyrosinase activity was assayed by measuring the L-3,4-dihydroxyphenylalanine (L-DOPA) oxidase activity using a previously described method [22]. The B16F10 cells were incubated with 100 μM 8-MOP and Isorhamnetin and Kaempferide (8, 16, 32 μM) for 24 h. At the end point of this treatment, the cells were washed twice with ice-cold PBS, lysed with 50 mM sodium phosphate buffer (pH 6.8) containing 1% Triton X-100 and phenylmethylsulfonyl fluoride (0.1 mM), and frozen at 80 °C for 30 min. Tyrosinase activity was analyzed spectrophotometrically. The dopachrome concentration in the reaction mixture was measured at 475 nm (Thermo Varioskan Flash 3001, USA). The reaction mixture containing 140 μL freshly prepared substrate solution (0.1% L-DOPA in 0.1 M sodium phosphate, pH 6.0) and 70 μL enzyme solution was incubated at 37 °C. The change in absorbance was measured for the first 2 h of the reaction. Corrections were made for the auto-oxidation of L-DOPA in the control. The tyrosinase activity was normalized against the protein content of the samples, which was determined using a commercial Bradford assay kit (Shanghai Sangong Co., China).

### Quantitative real-time PCR (qRT-PCR)

The total cellular RNA was isolated using a commercial kit (Shanghai Sangong Co., China). RNA quality was tested using the A260/A280 ratio and 1.5% agarose gel electrophoresis. The cDNA synthesis was performed using Moloney murine leukemia virus reverse transcriptase with a First Strand cDNA Synthesis Kit (Thermofisher, US). The cDNA synthesis system was performed according to the manufacturer’s instructions. The abundance of MC1R (241 bp), MITF (414 bp), TYR (223 bp), TYRP1 (116 bp), DCT (132 bp), and GAPDH (183 bp) mRNA in the samples were quantified using SYBR Green-based Rotor-Gene Q (Qiagen, German) and quantified using the 2^-ΔΔCt^ method. The mRNA expression was normalized using GAPDH as an endogenous control. The amplification was performed for 45 cycles (denaturing at 95 °C for 10 min, annealing at 95 °C for 5 s, and extension at 60 °C for 45 s). The primers were synthesized by Sangong Co. Ltd. (Shanghai, China). The forward and reverse primer sequences were as followed: 5′-GCTAAGGTCAGAGGGAGGGA-3′ and 5′-TCACCATAGAGGCACGAGGA-3′ for MC1R, 5′-CCCGCTTCTGGAAACTTGATCG-3′ and 5′-CTGTACTCTGAGCAGCAGGTG-3′ for MITF, 5′-TGACAAATGGCTGCGAAGGC-3′ and 5′-GGCTTGTTCCAAGTAAGGATC-3′ for TYR, 5′-TCAGGTTTGG GCTCAGTTTC-3′ and 5′-ATGAGCCACAAGGGTCAGTC-3′ for TYRP1, 5′-CGTG CTGAACAAGGAATGC-3′ and 5′-CGAAGGATATAAGGGCCACTC-3′ for DCT, 5′-GGTTGTCTCCTGCGACTTCA-3′ and 5′-TGGTCCAGGGTTTCTTACTCC-3′ for GAPDH.

### Western blot analyses and antibodies

Adherent cells were treated with 100 μM 8-MOP and Isorhamnetin and Kaempferide (8, 16, 32 μM) for 24 h, washed once with DPBS, and lysed with 1% NP-40 buffer (150 mM NaCl, 50 mM Tris, pH 7.5, 1 mM EDTA, and 1% NP-40) containing 1X protease inhibitors (Shanghai Sangong Co., China) and 1X phosphatase inhibitors (Shanghai Sangong Co., China). Lysates were quantified (Bradford assay), normalized, reduced, denatured (95 °C) and resolved by SDS gel electrophoresis on 12% Tris/Glycine gels (Shanghai Sangong Co., China). Resolved protein was transferred to PVDF membranes (Millipore, Billerica, MA) using a Semi-Dry Transfer Cell (Bio-Rad), blocked in 5% BSA in TBS-T and probed with primary antibodies recognizing MITF (Cell Signaling Technology, #12590, 1:1000, Danvers, MA) and β-Actin (Cell Signaling Technology, #3700, 1:4000). After incubation with the appropriate secondary antibody, proteins were detected using EC3™ 510 Imaging System Manual Platform (Ultra-Violet Products Ltd., Cambridge, UK).

### Statistical analysis

All data are expressed as mean ± standard error of the mean (SEM). The means, standard errors, and Student’s t-test results were performed using the SPSS 19.0 for Windows. *P* < 0.05 was considered statistically significant. All experiments were done at least 3 times with similar results.

## Results

### All compounds in *Vernonia anthelmintica (L.)* collecting and sorting

Forty-eight compounds were collected from *Vernonia anthelmintica (L.)* (Additional file 1: Table S1). These compounds may be divided into several main categories according to their structure, as follows: Steroids (18/48), Terpenes (13/48), Flavonoids (11/48), Fatty Acids (3/48), and others (3/48).

By evaluating the absorption associated properties of the compounds, 37 potential active compounds, which accounted for 77% of all compounds, were sorted according to their positive results evaluated by both the HIA and Caco-2 models (Additional file 2: Table S2). By evaluating the metabolism-associated properties of potential active compounds and the pharmacokinetic data sorting of active compounds, the top 5 active compounds were maintained as lead compounds for a further pharmacological mechanism study (Table 1; Additional file 2: Table S2). Among these compounds, Isorhamnetin and Kaempferide, which are methylated flavones, performed 1st and 2nd.

**Table 1:** The ADME properties *in silico* of top 5 compounds via admetSAR Prediction

Compounds	Absorption		Metabolism (CYP450 isoforms Inhibitor)					
	HIA	Caco-2	CYP450 1A2	CYP450 2C9	CYP450 2D6	CYP450 2C19	CYP450 3A4	*score*
Isorhamnetin	+0.9783	+0.8866	+0.9218	+0.7560	−0.6993	+0.8648	+0.7348	1.92963575
Kaempferide	+0.9783	+0.8866	+0.9218	+0.7560	−0.6993	+0.8648	+0.7348	1.92963575
Isoliquiritigenin	+0.9894	+0.8867	+0.935	+0.8949	−0.9231	+0.8994	+0.7959	1.92766477
Apigenin	+0.9887	+0.8541	+0.9222	+0.7746	−0.9231	+0.7043	+0.9580	1.76820103
Liquiritigenin	+0.9915	+0.7142	+0.8739	+0.9352	−0.8850	+0.8456	+0.5207	1.72194393

### Analysis of compound-target-pathway interactions

**Fig. 2:**
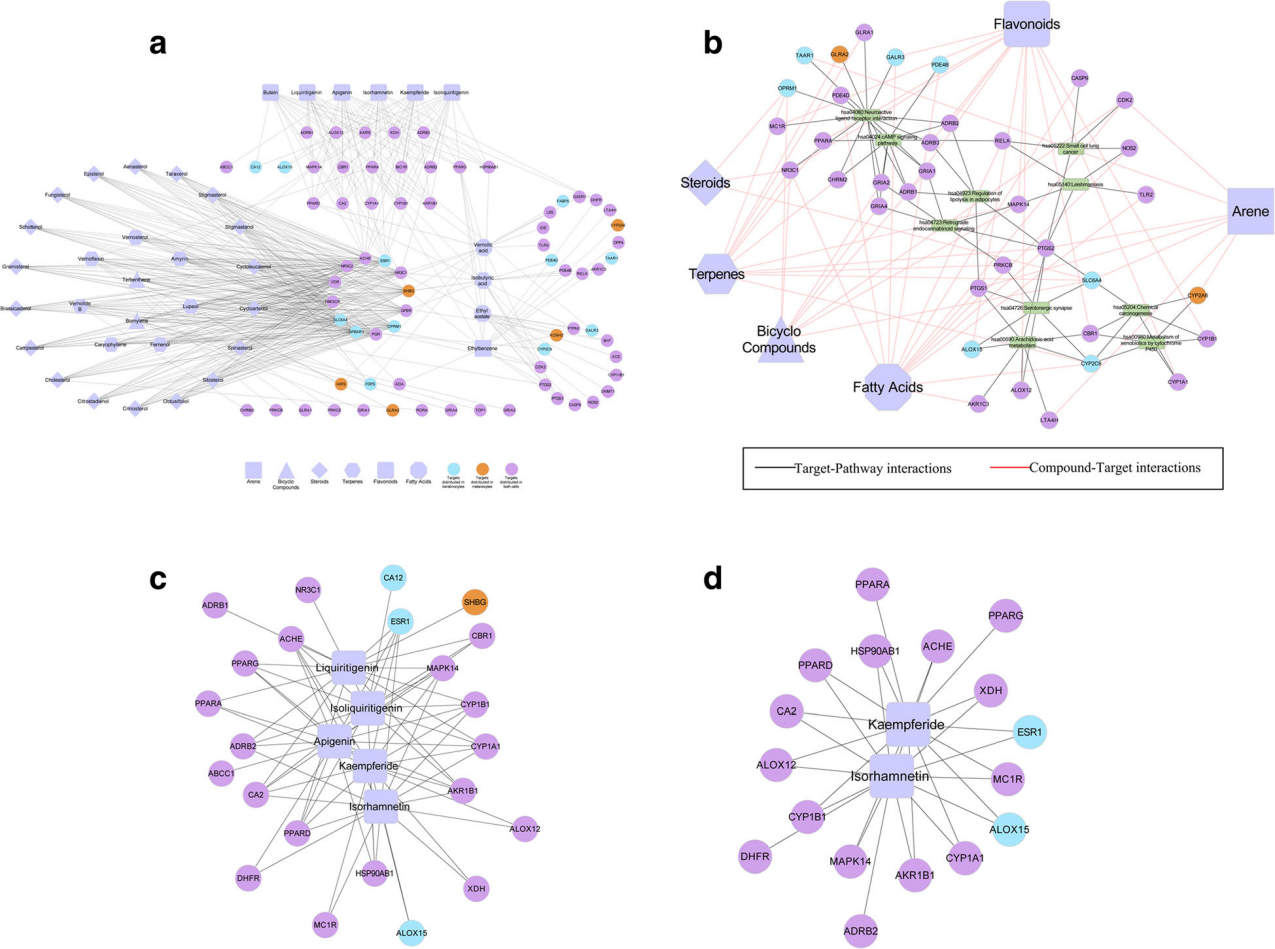
Compound-Target-Pathway interactions. By evaluating the absorption-associated properties of compounds, the 37 potential active compounds with 72 targets were used to construct the CTI network (**a**). The Potential Active Compound-Target and Pathway association network (**b**). By evaluating the metabolism-associated properties of compounds, the top 5 active compounds with 23 targets were used to construct the CTI network (**c**). Isorhamnetin and Kaempferide with 17 targets were used to construct the CTI network (**d**)

We obtained 102 candidate targets for the 37 potential active compounds with 713 connections between them using the SDTNBI method (Additional file 3: Table S3). After mapping the tissue-specific targets, we identified 72 targets related to keratinocytes and melanocytes, including 12 targets that are only expressed in keratinocytes and 5 targets that are only expressed in melanocytes (Additional file 4: Table S4). This tissue-specific problem had been ignored for a long time in previous studies. The 37 potential active compounds with 72 targets were subsequently used to construct the CTI network (Fig. 2a). We then performed the pathway analysis for the 72 candidate targets using the DAVID database (Additional file 5: Table S5). The results showed that the Neuroactive ligand-receptor interaction pathway, cAMP signaling pathway, arachidonic acid metabolism pathway and serotonergic synapses were related to flavonoids (Fig. 2b). Overall, 36.1% of the targets belonged to each pathway targeted by flavonoids and other compounds.

After the pharmacokinetic data sorting of active compounds, these top 5 active compounds with higher *scores* could be used as lead compounds in the treatment of vitiligo. These 5 active compounds with 23 targets were used to construct the CTI network (Fig. 2c). We subsequently performed the pathway analysis for these 23 targets using the DAVID database. The results included Arachidonic acid metabolism (ALOX12, ALOX15 and CBR1) and the PPAR signaling pathway (PPARA, PPARD and PPARG). Recent studies have indicated the cooperation between the PPAR family and the melanocyte-stimulating hormone receptor (MC1R)-microphthalmia-associated transcription factor (MITF) signaling pathway, which resulted in enhanced melanogenesis in melanocytes and melanoma cells. Furthermore, MC1R, which was predicted with Isorhamnetin (*Score =* 1.92963575) and Kaempferide (*Score =* 1.92963575) using SDTNBI, was related to the positive regulation of the cAMP biosynthetic process and the expression of melanin-biosynthetic genes. Isorhamnetin and Kaempferide along with 17 targets were subsequently used to construct the CTI network (Fig. 2d). Their were 13 common targets, which were ACHE, ALOX12, ALOX15, AKR1B1, CA2, CYP1A1, CYP1B1, ESR1, HSP90AB1, MAPK14, MC1R, PPARD, and XDH. Moreover, STRING identified a large potential interaction network between 56 vitiligo risk genetic loci and 13 common targets (Additional file 6: Figure S1) [23, 24]. We determined that MC1R was not only a vitiligo risk genetic locus but also was targeted by Isorhamnetin and Kaempferide.

Based on the admetSAR and SDTNBI methods, Isorhamnetin and Kaempferide from *Vernonia anthelmintica (L.)* were sorted and could be used as lead compounds in the treatment of vitiligo. To determine whether Isorhamnetin and Kaempferide increased melanin production, Isorhamnetin and Kaempferide were selected for further research.

### Effects of Isorhamnetin and Kaempferide on the viability of B16F10 cells

**Fig. 3:**
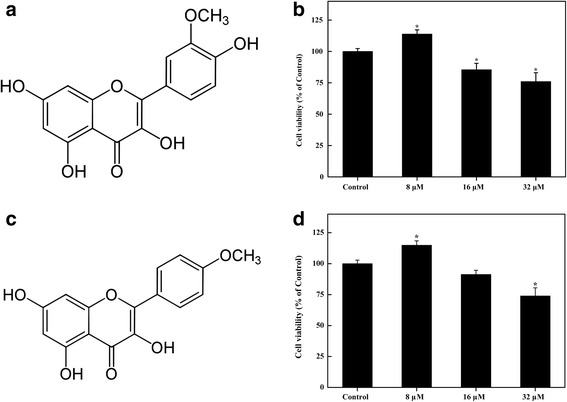
Effects on the cell viability of B16F10 melanoma cells following treatment with Isorhamnetin and Kaempferide. The structure of Isorhamnetin (**a**). Effects on the cell viability of B16F10 melanoma cells following treatment with Isorhamnetin (**b**). The structure of Kaempferide (**c**). Effects on the cell viability of B16F10 melanoma cells following treatment with Kaempferide (**d**). The B16F10 melanoma cells were incubated with medium that contained various concentrations (8–32 μM) for 24 h. Cell viability was determined using the MTT assay and is expressed as the means ± standard errors of at least 3 independent experiments performed in triplicate. ^*^ *P* < 0.05 vs. the control

The effects of Isorhamnetin and Kaempferide on the viability of B16F10 cells were examined using the MTT assay. Treatment with 8 μM Isorhamnetin and Kaempferide slightly stimulated B16F10 cell viability. Isorhamnetin and Kaempferide were not significantly cytotoxic to B16F10 cells at the concentrations of 16–32 μM for 24 h (Fig. 3b, d).

### Effects of Isorhamnetin and Kaempferide on melanin synthesis in B16F10 cells

**Fig. 4:**
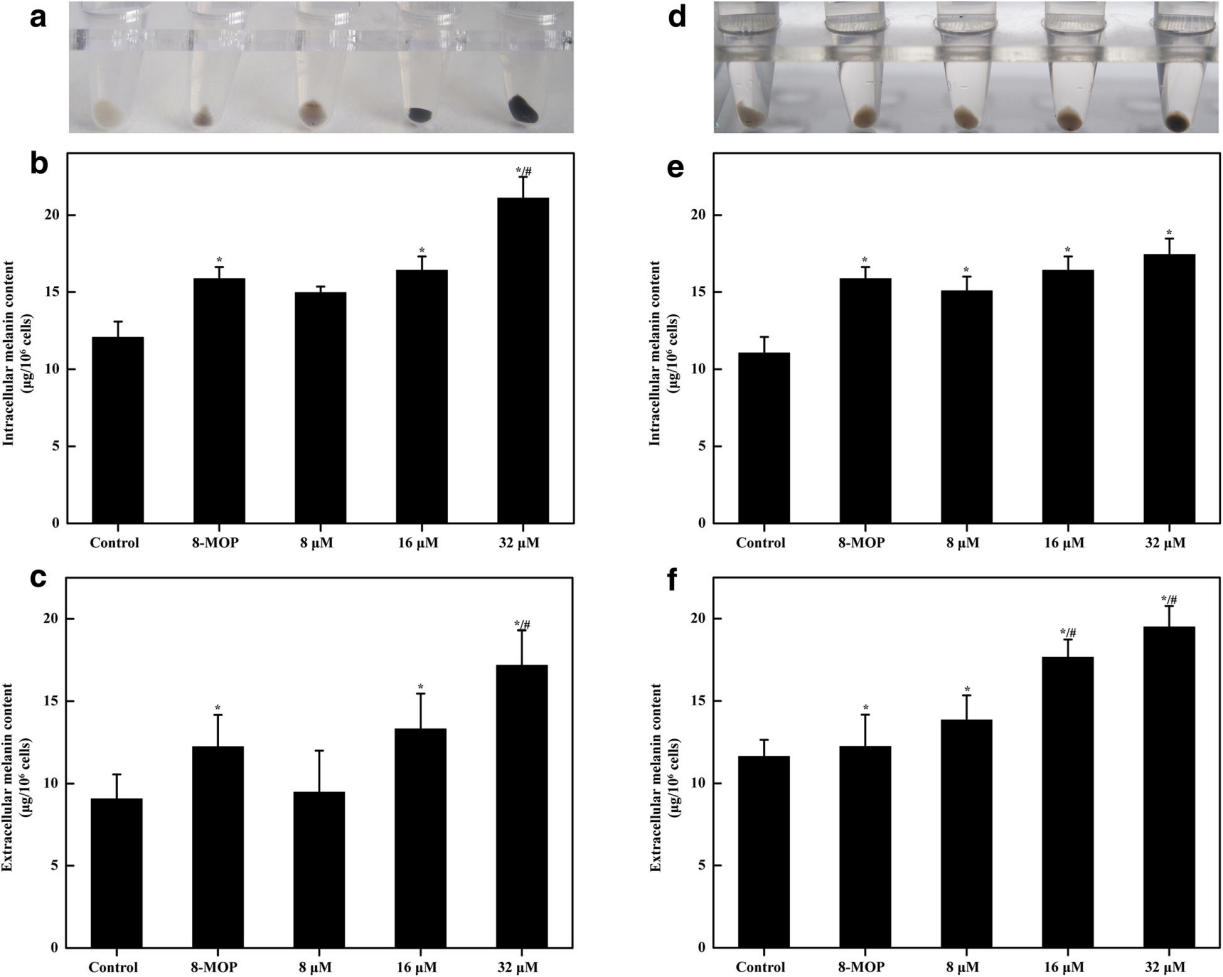
Effects of Isorhamnetin and Kaempferide on melanin production in B16F10 melanoma cells. Appearance of the recovered cell pellets in test tubes by Isorhamnetin (**a**). The intracellular melanin contents in Isorhamnetin-treated B16F10 melanoma cells for 24 h (**b**). Extracellular melanin contents in Isorhamnetin-treated B16F10 melanoma cells for 24 h (**c**). Appearance of the recovered cell pellets in test tubes by Kaempferide (**d**). Intracellular melanin contents in Kaempferide-treated B16F10 melanoma cells for 24 h (**e**). Extracellular melanin contents in Kaempferide-treated B16F10 melanoma cells for 24 h (**f**). Data shown represent the means ± standard error of at least 3 independent experiments performed in triplicate. ^*^ *P* < 0.05 vs. the control, ^#^ *P* < 0.05 vs. 8-MOP (100 μM)

To investigate the effects of Isorhamnetin and Kaempferide on melanin synthesis, B16F10 cells were exposed to Isorhamnetin and Kaempferide in a range from 0 to 32 μM for 24 h. Isorhamnetin significantly increased intracellular and extracellular melanin synthesis in a dose-dependent manner (Fig. 4a, b). Kaempferide also increased the intracellular and extracellular melanin synthesis in a dose-dependent manner (Fig. 4c, d). These results indicate that Isorhamnetin and Kaempferide have strong stimulating effects on melanin production by B16F10 cells.

### Effects of Isorhamnetin and Kaempferide on the melanogenic pathway in B16F10 cells

**Fig. 5:**
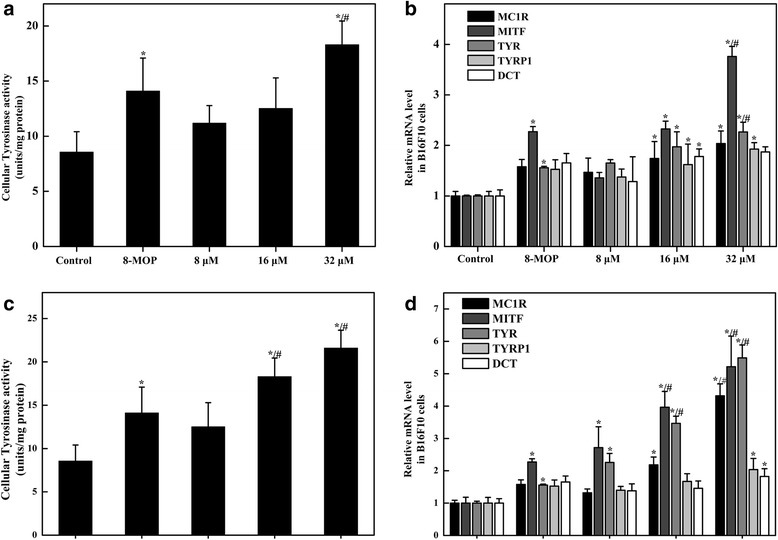
Effects of Isorhamnetin and Kaempferide on tyrosinase activity and melanin-biosynthetic genes in B16F10 melanoma cells. Effects of Isorhamnetin on tyrosinase activity in B16F10 melanoma cells (**a**). Quantified RT-PCR (QPCR) results in Isorhamnetin treatment via relative gene expression ratios to GAPDH (**b**). Effects of Kaempferide on tyrosinase activity in B16F10 melanoma cells (**c**). Quantified RT-PCR (QPCR) results in Kaempferide treatment via relative gene expression ratios to GAPDH (**d**). Data shown are the means ± standard errors of at least 3 independent experiments performed in triplicate. ^*^ *P* < 0.05 vs. the control, ^#^ *P* < 0.05 vs. 8-MOP (100 μM)

**Fig. 6:**
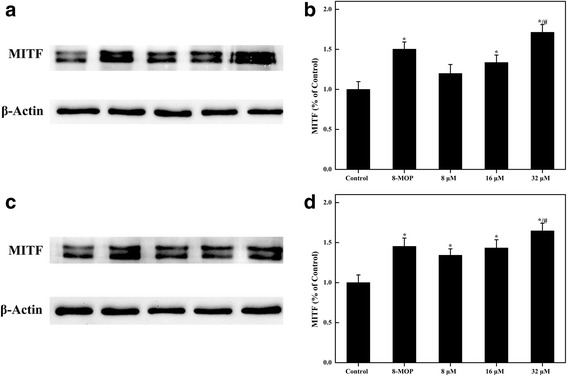
Effects of Isorhamnetin and Kaempferide on the protein level of MITF in B16F10 melanoma cells. Representative Western blot of MITF in Isorhamnetin-treated B16F10 melanoma cells for 24 h (**a**). Relative quantitative analysis of MITF in Isorhamnetin-treated B16F10 melanoma cells for 24 h (**b**). Representative Western blot of MITF in Kaempferide-treated B16F10 melanoma cells for 24 h (**c**). Relative quantitative analysis of MITF in Kaempferide-treated B16F10 melanoma cells for 24 h (**d**). Data shown are the means ± standard error of at least 3 independent experiments performed in triplicate. ^*^P < 0.05 vs. the control, ^#^P < 0.05 vs. 8-MOP (100 μM)

As Isorhamnetin and Kaempferide increased melanin synthesis, we further explored whether Isorhamnetin and Kaempferide affected tyrosinase activity, the expression of melanin-biosynthetic genes (MC1R, MITF, TYR, TYRP1 and DCT) and the protein level of MITF. Our data showed that Isorhamnetin and Kaempferide significantly increased tyrosinase activity in a dose-dependent manner (Fig. 5a, c). The mRNA expressions of MC1R, MITF, TYR, TYRP1 and DCT also increased by Isorhamnetin and Kaempferide treatment in a dose-dependent manner (Fig. 5b, d). Among these effects, Kaempferide induced the expression of more melanin-biosynthetic genes (MC1R, MITF and TYR) than Isorhamnetin. Furthermore, the protein level of MITF was increased via Isorhamnetin and Kaempferide treatment in a dose-dependent manner (Fig. 6). Thus, the melanogenic pathway was activated by Isorhamnetin and Kaempferide.

## Discussion

Vitiligo is the most common depigmentation skin disorder. It is caused by dysfunction or destruction of melanocytes, which are the main pigment-producing cells [25]. Although it is not physically harmful or contagious, vitiligo is often psychologically devastating. The available therapeutic treatments, including excimer laser, vitamin D analogs and steroid therapy, are unsuitable for many patients because they may be complex, time-consuming and ineffective [7]. Large-scale genome wide association studies, principally in European-derived white and Chinese individuals, have discovered approximately 56 different genetic loci that contribute to vitiligo risk, and some loci also contribute to autoimmunity, oxidative stress and the melanogenesis of melanocytes [23, 24]. Therefore, a better understanding of the underlying mechanisms should make the therapeutic treatments more-specific and more-effective.

TUM has been successfully used for the treatment of vitiligo in China, particularly *Vernonia anthelmintica (L.).* It has been reported that *Vernonia anthelmintica (L.)* injection has a significant therapeutic effect on vitiligo [26]. Moreover, the ethanol extract from *Vernonia anthelmintica (L.)* has been identified to enhance melanin synthesis by activating the p38 MAPK signaling pathway in B16F10 cells and primary melanocytes [27, 28]. Although previous studies have shown that *Vernonia anthelmintica (L.)* could promote melanogenesis, the key active compounds had not been investigated in depth. This issue hindered the modernization development of *Vernonia anthelmintica (L.)* for the treatment of vitiligo.

The admetSAR will be helpful for the in silico sorting of ADMET profiles of drug candidates and environmental chemicals. The admetSAR integrated high quality and predictive QSAR models to predict approximately 50 ADMET end-points, including HIA and Caco-2 permeability (Caco-2), as well as the inhibitors of CYP450 1A2, CYP450 2C9, CYP450 2C19 and CYP450 3A4. The area under the receiver operating characteristic curve (AUC) was used to evaluate the reliability of the predictions of 22 classification models via 5-fold cross validation [13].

Using admetSAR, the top 5 active compounds with positive absorption and a higher *score* could be used as potential lead compounds in treatment. These top 5 active compounds included Isoliquiritigenin, Apigenin, Liquiritigenin, Kaempferide and Isorhamnetin. Among these compounds, Kaempferide and Isorhamnetin, which are methylated flavones, exhibited the best parameters in absorption and metabolism. Dr. Thomas Walle has also proved that methylated flavones had substantially higher metabolic stability than those of the unmethylated forms [29]. Therefore, Isorhamnetin and Kaempferide were regarded as melanogenesis stimulators and potential agents for vitiligo.

In the early work of Si et al., Isorhamnetin (0–1500 μM) showed depigmentation activity via a biochemical tyrosinase-inhibitory experiment and a computational simulation approach [30]. However, additional pharmacodynamic evidence in vitro supported Isorhamnetin’s melanogenic activity. Maimaiti et al. have reported the melanogenic activity of Isorhamnetin at a concentration of 50,000 μM in a B16 cell line [8]. Ayako Kumagai et al. have reported the melanogenic activity of Isorhamnetin at a concentration of 10 μM in a B16F10 cell culture [31]. In the present study, we determined that Isorhamnetin (ranged from 0 to 32 μM) increased the tyrosinase activity and promoted melanogenesis in a B16F10 cell culture. The supporting binding data had showed Isorhamnetin and Kaempferide docking with tyrosinase to display their potential relevance (Additional file 7: Figure S2). Furthermore, an Isorhamnetin-similar compound, Kaempferide, displayed similar melanogenic effects. Based on three different experimental conditions and two similar compounds, we agree with the observation of the melanogenesis activity of Isorhamnetin.

Our pharmacological data in vitro showed that both Isorhamnetin and Kaempferide significantly increased the mRNA-expression of melanin-biosynthetic genes (MC1R, MITF, TYR, TYRP1 and DCT), as well as the protein level of MITF and the tyrosinase activity. All results supported the melanogenesis activity of Isorhamnetin. However, the melanogenic mechanisms of Isorhamnetin and Kaempferide have not yet been reported.

As a result of the rapid development of network pharmacology, the identification of new drug-target interactions (DTIs) will help investigators understand the molecular mechanisms of drugs and identify new uses for old drugs [32–34]. The SDTNBI method is a powerful network-based approach that predicts potential targets for potential active compounds on a large scale [11]. Systematic evaluation based on a 10-fold cross validation, leave-one-out cross validation and external validation showed the high accuracy and robustness of SDTNBI.

**Fig. 7:**
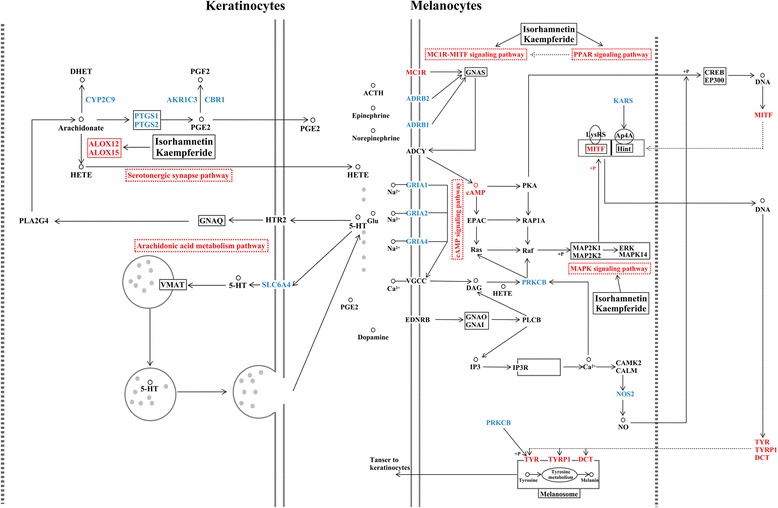
The melanogenic pathway of Isorhamnetin and Kaempferide from *Vernonia anthelmintica (L.)* for the treatment of vitiligo. The blue nodes represent the targets predicted by SDTNBI. The red nodes are closely related to melanin-biosynthetic genes and vitiligo pathogenesis

Based on the SDTNBI method and experimental verification, Isorhamnetin and Kaempferide effectively increased melanogenesis by targeting the MC1R-MITF signaling pathway, MAPK signaling pathway, PPAR signaling pathway (PPARA, PPARD, PPARG), arachidonic acid metabolism pathway (ALOX12, ALOX15, CBR1) and serotonergic synapses (ALOX12, ALOX15). We were more concerned that the MC1R activated adenylate cyclase and increased the cAMP level. Moreover, the secondary messenger cAMP activated a cascade of downstream transcriptional events, which led to the expression of melanin-biosynthetic genes (MITF, TYR, TYRP1, and DCT) [35]. The expression of tyrosinase and its related proteins (TYRP1 and DCT) was mainly regulated by MITF, which has previously been demonstrated to be important to the regulation of melanocyte differentiation, proliferation and survival [36]. A therapy for vitiligo must not only promote the repopulation of melanocytes but also increase melanogenesis. Zhou also reported that the transcription regulation of MITF was regulated by several signaling pathways, such as the MAPK signaling pathway [37]. In a previous study, Kang and co-workers investigated the expression and function of the PPAR signaling pathway, which belongs to the superfamily of nuclear receptors that heterodimerize with the retinoic X receptor and were initially known for their important role in melanocyte proliferation, differentiation and melanogenesis [38]. The arachidonic acid metabolism pathway and serotonergic synapses existed in keratinocytes, as well as pigment cells, which excreted regulative factors, such as PGE2, HETE and 5-HT. These regulative factors stimulated the proliferation of melanocytes and enhanced melanogenesis [39, 40]. The melanogenic pathway was activated by Isorhamnetin and Kaempferide (Fig. 7).

The melanogenic pathway based on the admetSAR and SDTNBI approaches was developed to identify the network pharmacological mechanisms of *Vernonia anthelmintica (L.)* for the treatment of vitiligo. Compared with traditional experimental assays, the systematic in silico approaches of admetSAR and SDTNBI have enabled us to rapidly identify potential lead compounds and CTIs. Further studies are required to validate whether the active compounds promote melanogenesis in vivo.

## Conclusions

In summary, the admetSAR and SDTNBI methods were combined to disclose the complex network pharmacological mechanism of *Vernonia anthelmintica (L.)* for vitiligo treatments in silico and in vitro, which provides a novel understanding of TUM for the treatment of vitiligo.

## Additional files

Additional file 1: Table S1. Chemical properties of 48 compounds from *Vernonia anthelmintica (L.)*. (DOC 54 kb)

Additional file 2: Table S2. The ADME properties in silico of 48 compounds from *Vernonia anthelmintica (L.)* via admetSAR Prediction. (DOC 166 kb)

Additional file 3: Table S3. The Compounds-Targets interaction information based on SDTNBI method. (DOC 1319 kb)

Additional file 4: Table S4. The information of tissue-specific targets. (DOC 144 kb) (DOC 144 kb)

Additional file 5: Table S5. The pathway analysis of 72 candidate targets via DAVID database. (DOC 51 kb)

Additional file 6: Figure S1. STRING identified a large potential interaction network between 56 vitiligo risk genetic loci and Isorhamnetin/Kaempferide with 13 common targets. The purple nodes represent the targets predicted by SDTNBI. The yellow nodes are closely related to vitiligo risk genetic loci. (TIFF 458 kb)

Additional file 7: Figure S2. Computational Docking Simulations of Binding between Tyrosinase structure and Isorhamnetin/Kaempferide/8-MOP. The three different crystal structures (PDB ID: 2y9x; 4p6r; 5i38) were used to simulate the 3D tyrosinase structure. The Yellow dotted line represented possible hydrogen-bonding interactions of Isorhamnetin/Kaempferide/8-MOP by the software SYBYL-X 2.0. (TIFF 1550 kb)

## Abbreviations

ADRB: beta adrenergic receptor
ALOX12: arachidonate 12-lipoxygenase, 12S–type
ALOX15: arachidonate 15-lipoxygenase
Caco-2: Caco-2 Permeability
CBR1: Carbonyl reductase [NADPH] 1
CTI: Compounds-Targets interaction
DCT: L-dopachrome tautomerase
DTI: drug-target interaction
HIA: Human Intestinal Absorption
HPA: Human Protein Atlas
L-DOPA: L-3,4-dihydroxyphenylalanine
MC1R: melanocyte-stimulating hormone receptor
MITF: microphthalmia-associated transcription factor
MTT: 3-(4,5-dimethyl-thiazol-2-yl)-2,5-diphenyl tetrazolium bromide
PPARA: Peroxisome proliferator-activated receptor alpha
PPARD: Peroxisome proliferator-activated receptor delta
PPARG: Peroxisome proliferator-activated receptor gamma
SDTNBI: substructure-drug-target network-based inference
TUM: Traditional Uyghur Medicine
TYR: Tyrosinase
TYRP1: Tyrosinase-related protein 1
