# Supplementary material for: Network pharmacological mechanisms of Vernonia anthelmintica (L.) in the treatment of vitiligo: Isorhamnetin induction of melanogenesis via up-regulation of melanin-biosynthetic genes
Source: BMC Syst Biol. 2017 Nov 16;11:103. doi: 10.1186/s12918-017-0486-1 (PMC5691595; doi:10.1186/s12918-017-0486-1)
Supplement: Supplementary file 1 — Chemical properties of 48 compounds from Vernonia anthelmintica (L.). (DOC 54 kb) [file 12918_2017_486_MOESM1_ESM.doc]

**Table S1. Chemical properties of 48 compounds from *Vernonia anthelmintica (L.)***

| **Compounds** | **Molecular Formula** | **MW** | **PubChem CID** | **Ancestors** |
| --- | --- | --- | --- | --- |
| Ethylbenzene | C_8_H_10_ | 106.168 | 7500 | Arene |
| Bornylene | C_10_H_16_ | 136.238 | 10047 | Bicyclo Compounds |
| Terbenthene | C_10_H_16_ | 136.238 | 14896 | Bicyclo Compounds |
| Isobutyric acid | C_4_H_8_O_2_ | 88.106 | 6590 | Fatty Acids |
| Ethyl acetate | C_4_H_8_O_2_ | 88.106 | 8857 | Fatty Acids |
| Vernolic acid | C_18_H_32_O_3_ | 296.451 | 6449780 | Fatty Acids |
| Liquiritigenin | C_15_H_12_O_4_ | 256.257 | 114829 | Flavonoids |
| Isoliquiritigenin | C_15_H_12_O_4_ | 256.257 | 638278 | Flavonoids |
| Apigenin | C_15_H_10_O_5_ | 270.24 | 5280443 | Flavonoids |
| Butin | C_15_H_12_O_5_ | 272.256 | 92775 | Flavonoids |
| Butein | C_15_H_12_O_5_ | 272.256 | 5281222 | Flavonoids |
| Kaempferol | C_15_H_10_O_6_ | 286.2363 | 5280863 | Flavonoids |
| Luteolin | C_15_H_10_O_6_ | 286.239 | 5280445 | Flavonoids |
| Scutellarein | C_15_H_10_O_6_ | 286.239 | 5281697 | Flavonoids |
| Eriodictyol | C_15_H_12_O_6_ | 288.255 | 440735 | Flavonoids |
| Kaempferide | C_16_H_12_O_6_ | 300.266 | 5281666 | Flavonoids |
| Isorhamnetin | C_16_H_12_O_7_ | 316.265 | 5281654 | Flavonoids |
| Vernosterol | C_29_H_46_O | 410.686 | 101967170 | Steroids |
| Cholesterol | C_27_H_46_O | 386.664 | 5997 | Steroids |
| Episterol | C_28_H_46_O | 398.675 | 23724571 | Steroids |
| Crinosterol | C_28_H_46_O | 398.675 | 5283660 | Steroids |
| Brassicasterol | C_28_H_46_O | 398.675 | 5281327 | Steroids |
| fungisterol | C_28_H_48_O | 400.691 | 5283646 | Steroids |
| campesterol | C_28_H_48_O | 400.691 | 173183 | Steroids |
| Gramisterol | C_29_H_48_O | 412.702 | 5283640 | Steroids |
| Aenasterol | C_29_H_48_O | 412.702 | 71587058 | Steroids |
| Stigmasterol | C_29_H_48_O | 412.702 | 5280794 | Steroids |
| Spinasterol | C_29_H_48_O | 412.702 | 5281331 | Steroids |
| Sitosterol | C_29_H_50_O | 414.718 | 222284 | Steroids |
| Schottenol | C_29_H_50_O | 414.718 | 441837 | Steroids |
| Stigmastanol | C_29_H_52_O | 416.734 | 15559396 | Steroids |
| Obtusifoliol | C_30_H_50_O | 426.729 | 65252 | Steroids |
| Cycloleucalenol | C_30_H_50_O | 426.729 | 101690 | Steroids |
| Cycloartenol | C_30_H_50_O | 426.729 | 17750995 | Steroids |
| Citrostadienol | C_30_H_50_O | 426.729 | 9548595 | Steroids |
| Amyrin | C_30_H_50_O | 426.729 | 73145 | Terpenes |
| Taraxerol | C_30_H_50_O | 426.729 | 92097 | Terpenes |
| Lupeol | C_30_H_50_O | 426.729 | 259846 | Terpenes |
| Fernenol | C_30_H_50_O | 426.729 | 12305178 | Terpenes |
| Caryophyllene | C_15_H_24_ | 204.357 | 5281522 | Terpenes |
| Vernolepin | C_15_H_16_O_5_ | 276.288 | 442322 | Terpenes |
| Vernoflexin | C_20_H_24_O_4_ | 328.408 | 442319 | Terpenes |
| Vernodalin | C_19_H_20_O_7_ | 360.362 | 179375 | Terpenes |
| Vernolide A | C_21_H_28_O_7_ | 392.448 | 70690655 | Terpenes |
| Vernolide B | C_23_H_30_O_8_ | 434.485 | 70692803 | Terpenes |
| Vernolide C | C_21_H_27_ClO_9_ | 458.888 | 101412351 | Terpenes |
| Vernolide D | C_22_H_28_O_9_ | 436.457 | 101412352 | Terpenes |
| vernodalol | C_20_H_24_O_8_ | 392.404 | 442318 | Terpenes |
