# Supplementary material for: Network pharmacological mechanisms of Vernonia anthelmintica (L.) in the treatment of vitiligo: Isorhamnetin induction of melanogenesis via up-regulation of melanin-biosynthetic genes
Source: BMC Syst Biol. 2017 Nov 16;11:103. doi: 10.1186/s12918-017-0486-1 (PMC5691595; doi:10.1186/s12918-017-0486-1)
Supplement: Supplementary file 2 — The ADME properties in silico of 48 compounds from Vernonia anthelmintica (L.) via admetSAR Prediction. (DOC 166 kb) [file 12918_2017_486_MOESM2_ESM.doc]

**Table S2. The ADME properties *in silico* of 48 compounds from *Vernonia anthelmintica (L.)* via admetSAR Prediction**

| **Number** | **Compounds** | **Absorption** | |  | **Metabolism (CYP450 isoforms Inhibitor)** | | | | | |
| --- | --- | --- | --- | --- | --- | --- | --- | --- | --- | --- |
|  |  | **HIA** | **Caco-2** |  | **CYP450 1A2** | **CYP450 2C9** | **CYP450 2D6** | **CYP450 2C19** | **CYP450 3A4** | ***score*** |
| 1 | Isorhamnetin | +0.9783 | +0.8866 |  | +0.9218 | +0.756 | -0.6993 | +0.8648 | +0.7348 | 1.92963575 |
| 2 | Kaempferide | +0.9783 | +0.8866 |  | +0.9218 | +0.756 | -0.6993 | +0.8648 | +0.7348 | 1.92963575 |
| 3 | Isoliquiritigenin | +0.9894 | +0.8867 |  | +0.935 | +0.8949 | -0.9231 | +0.8994 | +0.7959 | 1.92766477 |
| 4 | Apigenin | +0.9887 | +0.8541 |  | +0.9222 | +0.7746 | -0.9231 | +0.7043 | +0.958 | 1.76820103 |
| 5 | Liquiritigenin | +0.9915 | +0.7142 |  | +0.8739 | +0.9352 | -0.885 | +0.8456 | +0.5207 | 1.72194393 |
| 6 | Kaempferol | +0.9855 | -0.7447 |  | +0.9108 | +0.8948 | -0.9083 | +0.6434 | +0.7241 | 1.66803093 |
| 7 | Eriodictyol | +0.9223 | -0.8576 |  | +0.8188 | +0.5787 | -0.7926 | -0.7926 | +0.7009 | 0.26704622 |
| 8 | Butein | +0.9704 | +0.6078 |  | +0.8928 | +0.5111 | -0.9263 | -0.8768 | +0.6787 | 0.07667179 |
| 9 | Butin | +0.9795 | -0.8199 |  | +0.7993 | +0.8265 | -0.9044 | -0.6997 | -0.7378 | -0.49889441 |
| 10 | Luteolin | +0.965 | -0.8957 |  | +0.9106 | -0.5823 | -0.9287 | -0.9025 | +0.6951 | -0.79768769 |
| 11 | Scutellarein | +0.965 | -0.8957 |  | +0.9106 | -0.5823 | -0.9287 | -0.9025 | +0.6951 | -0.79768769 |
| 12 | Caryophyllene | +0.9926 | +0.6327 |  | -0.6695 | -0.6249 | -0.9284 | -0.5957 | -0.8665 | -2.87903059 |
| 13 | Vernolic acid | +0.9937 | +0.6558 |  | -0.5957 | -0.824 | -0.939 | -0.7613 | -0.7256 | -3.03010191 |
| 14 | Vernoflexin | +0.9702 | +0.5629 |  | -0.5831 | -0.8378 | -0.9011 | -0.7295 | -0.8365 | -3.04441202 |
| 15 | Vernodalin | +0.8035 | -0.5953 |  | -0.8615 | -0.849 | -0.9021 | -0.7117 | -0.5816 | -3.10231314 |
| 16 | Vernolide C | +0.9594 | -0.7356 |  | -0.7512 | -0.7702 | -0.9231 | -0.8319 | -0.6477 | -3.10667057 |
| 17 | Vernodalol | -0.6294 | +0.5452 |  | -0.8158 | -0.8075 | -0.9162 | -0.6844 | -0.7506 | -3.13088114 |
| 18 | Cycloleucalenol | +0.9935 | +0.7941 |  | -0.8045 | -0.7064 | -0.9424 | -0.7285 | -0.8015 | -3.13136531 |
| 19 | Cycloartenol | +0.9973 | +0.7777 |  | -0.7988 | -0.693 | -0.9443 | -0.7343 | -0.8212 | -3.13497991 |
| 20 | Terbenthene | +0.9834 | +0.6648 |  | -0.8077 | -0.75 | -0.9179 | -0.6602 | -0.8825 | -3.14521706 |
| 21 | Vernolide D | +0.9258 | -0.7598 |  | -0.7489 | -0.8277 | -0.95 | -0.9112 | -0.5236 | -3.15772617 |
| 22 | Vernolide A | +0.9448 | -0.5315 |  | -0.6935 | -0.8509 | -0.9569 | -0.9354 | -0.5762 | -3.19051142 |
| 23 | Amyrin | +1 | +0.831 |  | -0.8575 | -0.7983 | -0.9399 | -0.6636 | -0.8699 | -3.23793962 |
| 24 | Taraxerol | +1 | +0.831 |  | -0.8575 | -0.7983 | -0.9399 | -0.6636 | -0.8699 | -3.23793962 |
| 25 | Stigmastanol | +1 | +0.8027 |  | -0.7423 | -0.7611 | -0.9574 | -0.8361 | -0.8684 | -3.26718747 |
| 26 | Vernolepin | +0.8757 | -0.5294 |  | -0.8834 | -0.8173 | -0.8988 | -0.839 | -0.706 | -3.2746816 |
| 27 | Vernolide B | +0.9754 | +0.5089 |  | -0.7051 | -0.8494 | -0.9529 | -0.9191 | -0.757 | -3.29882682 |
| 28 | Lupeol | +0.9974 | +0.8499 |  | -0.8619 | -0.82 | -0.9506 | -0.732 | -0.8441 | -3.30652129 |
| 29 | Ethylbenzene | +0.9973 | +0.8969 |  | -0.554 | -0.8849 | -0.9423 | -0.904 | -0.9568 | -3.31183495 |
| 30 | Fernenol | +1 | +0.8343 |  | -0.8641 | -0.833 | -0.9446 | -0.7211 | -0.9059 | -3.34468857 |
| 31 | Schottenol | +1 | +0.8147 |  | -0.9019 | -0.8613 | -0.9292 | -0.7651 | -0.8031 | -3.35413823 |
| 32 | Spinasterol | +1 | +0.8147 |  | -0.9019 | -0.8613 | -0.9292 | -0.7651 | -0.8031 | -3.35413823 |
| 33 | Episterol | +0.9964 | +0.8566 |  | -0.9054 | -0.8492 | -0.946 | -0.748 | -0.8499 | -3.37806724 |
| 34 | Gramisterol | +0.9964 | +0.8566 |  | -0.9054 | -0.8492 | -0.946 | -0.748 | -0.8499 | -3.37806724 |
| 35 | Obtusifoliol | +0.9964 | +0.8566 |  | -0.9054 | -0.8492 | -0.946 | -0.748 | -0.8499 | -3.37806724 |
| 36 | Aenasterol | +1 | +0.8378 |  | -0.9088 | -0.8612 | -0.9483 | -0.7681 | -0.864 | -3.41770859 |
| 37 | Citrostadienol | +1 | +0.8378 |  | -0.9088 | -0.8612 | -0.9483 | -0.7681 | -0.864 | -3.41770859 |
| 38 | Fungisterol | +1 | +0.8378 |  | -0.9088 | -0.8612 | -0.9483 | -0.7681 | -0.864 | -3.41770859 |
| 39 | Vernosterol | +1 | +0.8378 |  | -0.9088 | -0.8612 | -0.9483 | -0.7681 | -0.864 | -3.41770859 |
| 40 | Ethyl acetate | +0.9955 | +0.7286 |  | -0.6609 | -0.9386 | -0.9531 | -0.9506 | -0.9801 | -3.50377826 |
| 41 | Sitosterol | +1 | +0.7953 |  | -0.9291 | -0.9125 | -0.9346 | -0.9025 | -0.8309 | -3.55056073 |
| 42 | Stigmasterol | +1 | +0.7953 |  | -0.9291 | -0.9125 | -0.9346 | -0.9025 | -0.8309 | -3.55056073 |
| 43 | Bornylene | +0.9969 | +0.7355 |  | -0.8575 | -0.9255 | -0.9601 | -0.9059 | -0.9122 | -3.57963352 |
| 44 | Brassicasterol | +1 | +0.8165 |  | -0.914 | -0.9071 | -0.9478 | -0.9025 | -0.8916 | -3.58436786 |
| 45 | Crinosterol | +1 | +0.8165 |  | -0.914 | -0.9071 | -0.9478 | -0.9025 | -0.8916 | -3.58436786 |
| 46 | Campesterol | +1 | +0.8184 |  | -0.9355 | -0.9194 | -0.9519 | -0.9177 | -0.8638 | -3.60956304 |
| 47 | Cholesterol | +1 | +0.8184 |  | -0.9355 | -0.9194 | -0.9519 | -0.9177 | -0.8638 | -3.60956304 |
| 48 | Isobutyric acid | +0.9848 | +0.598 |  | -0.95 | -0.9496 | -0.9649 | -0.9886 | -0.9818 | -3.78991971 |
