# Supplementary material for: Network pharmacological mechanisms of Vernonia anthelmintica (L.) in the treatment of vitiligo: Isorhamnetin induction of melanogenesis via up-regulation of melanin-biosynthetic genes
Source: BMC Syst Biol. 2017 Nov 16;11:103. doi: 10.1186/s12918-017-0486-1 (PMC5691595; doi:10.1186/s12918-017-0486-1)
Supplement: Supplementary file 3 — The Compounds-Targets interaction information based on SDTNBI method. (DOC 1319 kb) [file 12918_2017_486_MOESM3_ESM.doc]

**Table S3. The Compounds-Targets interaction information based on SDTNBI method**

| **Compound name** | **Rank** | **Score** | **Target** | **Uniprot ID** |
| --- | --- | --- | --- | --- |
| Aenasterol | 17 | 0.000203741 | ESR2 | Q92731 |
| Aenasterol | 3 | 0.000357074 | GPBAR1 | Q8TDU6 |
| Aenasterol | 13 | 0.000214053 | SLC10A2 | Q12908 |
| Aenasterol | 7 | 0.000283496 | RORC | P51449 |
| Aenasterol | 12 | 0.000217041 | OPRM1 | P35372 |
| Aenasterol | 16 | 0.000204192 | SLC6A4 | P31645 |
| Aenasterol | 14 | 0.000212752 | SLC6A2 | P23975 |
| Aenasterol | 11 | 0.000217388 | CYP19A1 | P11511 |
| Aenasterol | 4 | 0.000339068 | VDR | P11473 |
| Aenasterol | 5 | 0.0003094 | GPER1 | P10275 |
| Aenasterol | 15 | 0.000208194 | NR3C2 | P08235 |
| Aenasterol | 10 | 0.000256393 | PGR | P06401 |
| Aenasterol | 1 | 0.000733402 | SHBG | P04278 |
| Aenasterol | 2 | 0.000601256 | NR3C1 | P04150 |
| Aenasterol | 8 | 0.000274694 | HMGCR | P04035 |
| Aenasterol | 9 | 0.000269426 | ESR1 | P03372 |
| Aenasterol | 6 | 0.0003057 | RBP4 | P02753 |
| Aenasterol | 18 | 0.00019179 | GGPS1 | O95749 |
| Amyrin | 15 | 0.00020109 | ESR2 | Q92731 |
| Amyrin | 4 | 0.000319654 | GPBAR1 | Q8TDU6 |
| Amyrin | 17 | 0.000193018 | SLC10A2 | Q12908 |
| Amyrin | 9 | 0.00025301 | RORC | P51449 |
| Amyrin | 19 | 0.000180881 | OPRK1 | P41145 |
| Amyrin | 11 | 0.000224917 | OPRM1 | P35372 |
| Amyrin | 16 | 0.000200599 | SLC6A4 | P31645 |
| Amyrin | 14 | 0.000204226 | SLC6A2 | P23975 |
| Amyrin | 20 | 0.000176995 | ACHE | P22303 |
| Amyrin | 12 | 0.000214992 | CYP19A1 | P11511 |
| Amyrin | 6 | 0.000309779 | VDR | P11473 |
| Amyrin | 5 | 0.000310151 | GPER1 | P10275 |
| Amyrin | 13 | 0.000204498 | NR3C2 | P08235 |
| Amyrin | 8 | 0.000259561 | PGR | P06401 |
| Amyrin | 1 | 0.000709688 | SHBG | P04278 |
| Amyrin | 2 | 0.000598243 | NR3C1 | P04150 |
| Amyrin | 10 | 0.000230594 | HMGCR | P04035 |
| Amyrin | 7 | 0.000265675 | ESR1 | P03372 |
| Amyrin | 3 | 0.000322712 | RBP4 | P02753 |
| Amyrin | 18 | 0.00019179 | GGPS1 | O95749 |
| Apigenin | 3 | 0.000538675 | ESR2 | Q92731 |
| Apigenin | 6 | 0.000401108 | CYP1B1 | Q16678 |
| Apigenin | 12 | 0.000251374 | MAPK14 | Q16539 |
| Apigenin | 18 | 0.000204887 | PPARA | Q07869 |
| Apigenin | 2 | 0.000680752 | TAS2R31 | P59538 |
| Apigenin | 17 | 0.000214484 | PPARG | P37231 |
| Apigenin | 20 | 0.00019217 | ABCC1 | P33527 |
| Apigenin | 11 | 0.000251481 | ACHE | P22303 |
| Apigenin | 9 | 0.000261291 | CBR1 | P16152 |
| Apigenin | 4 | 0.000499761 | AKR1B1 | P15121 |
| Apigenin | 10 | 0.000253914 | HSD17B1 | P14061 |
| Apigenin | 7 | 0.000375382 | CYP19A1 | P11511 |
| Apigenin | 5 | 0.0004444 | PPARD | P11309 |
| Apigenin | 15 | 0.000222428 | HSP90AB1 | P08238 |
| Apigenin | 19 | 0.000198473 | ADRB2 | P07550 |
| Apigenin | 8 | 0.000303295 | CYP1A1 | P04798 |
| Apigenin | 1 | 0.000684987 | ESR1 | P03372 |
| Apigenin | 14 | 0.000236774 | CA2 | P00918 |
| Apigenin | 13 | 0.000248162 | CA1 | P00915 |
| Apigenin | 16 | 0.000219312 | DHFR | P00374 |
| Bornylene | 19 | 0.000185934 | ESR2 | Q92731 |
| Bornylene | 10 | 0.000248035 | GPBAR1 | Q8TDU6 |
| Bornylene | 13 | 0.000200166 | RORC | P51449 |
| Bornylene | 8 | 0.000249681 | GRIA4 | P48058 |
| Bornylene | 7 | 0.000249681 | GRIA2 | P42262 |
| Bornylene | 12 | 0.000240571 | GRIA1 | P42261 |
| Bornylene | 14 | 0.000199742 | OPRM1 | P35372 |
| Bornylene | 17 | 0.000191862 | SLC6A4 | P31645 |
| Bornylene | 15 | 0.0001965 | SLC6A2 | P23975 |
| Bornylene | 16 | 0.000192858 | ACHE | P22303 |
| Bornylene | 9 | 0.000248645 | CYP19A1 | P11511 |
| Bornylene | 4 | 0.000364012 | VDR | P11473 |
| Bornylene | 5 | 0.000293827 | GPER1 | P10275 |
| Bornylene | 18 | 0.000189441 | NR3C2 | P08235 |
| Bornylene | 6 | 0.000277268 | PGR | P06401 |
| Bornylene | 1 | 0.000693682 | SHBG | P04278 |
| Bornylene | 2 | 0.000541203 | NR3C1 | P04150 |
| Bornylene | 11 | 0.000244487 | ESR1 | P03372 |
| Bornylene | 3 | 0.000458682 | RBP4 | P02753 |
| Brassicasterol | 13 | 0.000208229 | ESR2 | Q92731 |
| Brassicasterol | 3 | 0.000343296 | GPBAR1 | Q8TDU6 |
| Brassicasterol | 17 | 0.00019978 | SLC10A2 | Q12908 |
| Brassicasterol | 7 | 0.000274369 | RORC | P51449 |
| Brassicasterol | 14 | 0.000205238 | OPRM1 | P35372 |
| Brassicasterol | 16 | 0.000200025 | SLC6A4 | P31645 |
| Brassicasterol | 18 | 0.000199626 | SLC6A2 | P23975 |
| Brassicasterol | 19 | 0.000188048 | ACHE | P22303 |
| Brassicasterol | 11 | 0.000215313 | CYP19A1 | P11511 |
| Brassicasterol | 6 | 0.000279495 | VDR | P11473 |
| Brassicasterol | 4 | 0.000300136 | GPER1 | P10275 |
| Brassicasterol | 15 | 0.000201888 | NR3C2 | P08235 |
| Brassicasterol | 9 | 0.00023801 | PGR | P06401 |
| Brassicasterol | 1 | 0.000678622 | SHBG | P04278 |
| Brassicasterol | 2 | 0.000579596 | NR3C1 | P04150 |
| Brassicasterol | 5 | 0.000283198 | HMGCR | P04035 |
| Brassicasterol | 8 | 0.000269704 | ESR1 | P03372 |
| Brassicasterol | 10 | 0.000228977 | RBP4 | P02753 |
| Brassicasterol | 12 | 0.000214701 | GGPS1 | O95749 |
| Butein | 2 | 0.000623436 | ESR2 | Q92731 |
| Butein | 14 | 0.000284658 | CYP1B1 | Q16678 |
| Butein | 18 | 0.000222878 | KARS | Q15046 |
| Butein | 4 | 0.000406152 | TAS2R31 | P59538 |
| Butein | 19 | 0.000216667 | ACHE | P22303 |
| Butein | 12 | 0.000298225 | ALOX12 | P18054 |
| Butein | 15 | 0.000250682 | CBR1 | P16152 |
| Butein | 3 | 0.000583426 | AKR1B1 | P15121 |
| Butein | 16 | 0.000247744 | ADRB3 | P13945 |
| Butein | 5 | 0.000403499 | CYP19A1 | P11511 |
| Butein | 6 | 0.000369105 | PPARD | P11309 |
| Butein | 9 | 0.000319363 | HSP90AB1 | P08238 |
| Butein | 10 | 0.000311604 | HSP90AA1 | P07900 |
| Butein | 7 | 0.000345571 | ADRB2 | P07550 |
| Butein | 17 | 0.000229159 | CYP1A1 | P04798 |
| Butein | 1 | 0.000876537 | ESR1 | P03372 |
| Butein | 11 | 0.000304015 | CA2 | P00918 |
| Butein | 8 | 0.000335211 | CA1 | P00915 |
| Butein | 13 | 0.00028716 | CA12 | O43570 |
| Campesterol | 13 | 0.000208677 | ESR2 | Q92731 |
| Campesterol | 3 | 0.000352133 | GPBAR1 | Q8TDU6 |
| Campesterol | 14 | 0.000207894 | SLC10A2 | Q12908 |
| Campesterol | 5 | 0.000283489 | RORC | P51449 |
| Campesterol | 15 | 0.000207617 | OPRM1 | P35372 |
| Campesterol | 16 | 0.000207127 | SLC6A4 | P31645 |
| Campesterol | 12 | 0.000209007 | SLC6A2 | P23975 |
| Campesterol | 19 | 0.000186212 | ACHE | P22303 |
| Campesterol | 10 | 0.000212607 | CYP19A1 | P11511 |
| Campesterol | 6 | 0.000276345 | VDR | P11473 |
| Campesterol | 4 | 0.000298953 | GPER1 | P10275 |
| Campesterol | 18 | 0.00020113 | NR3C2 | P08235 |
| Campesterol | 9 | 0.000237742 | PGR | P06401 |
| Campesterol | 1 | 0.000681007 | SHBG | P04278 |
| Campesterol | 2 | 0.000573305 | NR3C1 | P04150 |
| Campesterol | 7 | 0.00027283 | HMGCR | P04035 |
| Campesterol | 8 | 0.000270629 | ESR1 | P03372 |
| Campesterol | 11 | 0.000212136 | RBP4 | P02753 |
| Campesterol | 17 | 0.000203297 | GGPS1 | O95749 |
| Caryophyllene | 19 | 0.000171751 | ESR2 | Q92731 |
| Caryophyllene | 15 | 0.000202468 | GPBAR1 | Q8TDU6 |
| Caryophyllene | 18 | 0.000175203 | MAPK14 | Q16539 |
| Caryophyllene | 20 | 0.000166569 | RORC | P51449 |
| Caryophyllene | 12 | 0.000214196 | OPRK1 | P41145 |
| Caryophyllene | 9 | 0.000216701 | OPRD1 | P41143 |
| Caryophyllene | 5 | 0.000254884 | OPRM1 | P35372 |
| Caryophyllene | 7 | 0.000219412 | SLC6A4 | P31645 |
| Caryophyllene | 14 | 0.000210219 | SLC6A2 | P23975 |
| Caryophyllene | 16 | 0.000194925 | ACHE | P22303 |
| Caryophyllene | 13 | 0.00021105 | CYP19A1 | P11511 |
| Caryophyllene | 17 | 0.000190593 | VDR | P11473 |
| Caryophyllene | 4 | 0.000256805 | GPER1 | P10275 |
| Caryophyllene | 6 | 0.000248593 | ADH1A | P07327 |
| Caryophyllene | 10 | 0.000216475 | PGR | P06401 |
| Caryophyllene | 1 | 0.000532039 | SHBG | P04278 |
| Caryophyllene | 2 | 0.000468548 | NR3C1 | P04150 |
| Caryophyllene | 8 | 0.000219193 | ESR1 | P03372 |
| Caryophyllene | 3 | 0.000282575 | RBP4 | P02753 |
| Caryophyllene | 11 | 0.000215129 | GGPS1 | O95749 |
| Cholesterol | 15 | 0.000200872 | ESR2 | Q92731 |
| Cholesterol | 4 | 0.0003322 | GPBAR1 | Q8TDU6 |
| Cholesterol | 18 | 0.000196126 | SLC10A2 | Q12908 |
| Cholesterol | 3 | 0.000538635 | RORC | P51449 |
| Cholesterol | 10 | 0.000244984 | RORA | P35398 |
| Cholesterol | 17 | 0.000198618 | OPRM1 | P35372 |
| Cholesterol | 13 | 0.000203301 | SLC6A4 | P31645 |
| Cholesterol | 9 | 0.000257405 | SLC6A2 | P23975 |
| Cholesterol | 16 | 0.000200823 | CYP19A1 | P11511 |
| Cholesterol | 6 | 0.000263862 | VDR | P11473 |
| Cholesterol | 5 | 0.000282031 | GPER1 | P10275 |
| Cholesterol | 19 | 0.000189745 | NR3C2 | P08235 |
| Cholesterol | 11 | 0.000224285 | PGR | P06401 |
| Cholesterol | 1 | 0.000643069 | SHBG | P04278 |
| Cholesterol | 2 | 0.000540854 | NR3C1 | P04150 |
| Cholesterol | 8 | 0.000257695 | HMGCR | P04035 |
| Cholesterol | 7 | 0.000259402 | ESR1 | P03372 |
| Cholesterol | 12 | 0.000204384 | RBP4 | P02753 |
| Cholesterol | 14 | 0.000200944 | GGPS1 | O95749 |
| Citrostadienol | 14 | 0.000212378 | ESR2 | Q92731 |
| Citrostadienol | 3 | 0.000332196 | GPBAR1 | Q8TDU6 |
| Citrostadienol | 17 | 0.000200394 | SLC10A2 | Q12908 |
| Citrostadienol | 8 | 0.000266971 | RORC | P51449 |
| Citrostadienol | 11 | 0.000225373 | OPRM1 | P35372 |
| Citrostadienol | 16 | 0.000204615 | SLC6A4 | P31645 |
| Citrostadienol | 15 | 0.000210543 | SLC6A2 | P23975 |
| Citrostadienol | 19 | 0.000180242 | ACHE | P22303 |
| Citrostadienol | 12 | 0.000218137 | CYP19A1 | P11511 |
| Citrostadienol | 5 | 0.000315907 | VDR | P11473 |
| Citrostadienol | 4 | 0.000318312 | GPER1 | P10275 |
| Citrostadienol | 13 | 0.000213477 | NR3C2 | P08235 |
| Citrostadienol | 9 | 0.000260266 | PGR | P06401 |
| Citrostadienol | 1 | 0.000738785 | SHBG | P04278 |
| Citrostadienol | 2 | 0.000617352 | NR3C1 | P04150 |
| Citrostadienol | 10 | 0.0002373 | HMGCR | P04035 |
| Citrostadienol | 7 | 0.000279632 | ESR1 | P03372 |
| Citrostadienol | 6 | 0.0003057 | RBP4 | P02753 |
| Citrostadienol | 18 | 0.00019179 | GGPS1 | O95749 |
| Crinosterol | 13 | 0.000208229 | ESR2 | Q92731 |
| Crinosterol | 3 | 0.000343296 | GPBAR1 | Q8TDU6 |
| Crinosterol | 17 | 0.00019978 | SLC10A2 | Q12908 |
| Crinosterol | 7 | 0.000274369 | RORC | P51449 |
| Crinosterol | 14 | 0.000205238 | OPRM1 | P35372 |
| Crinosterol | 16 | 0.000200025 | SLC6A4 | P31645 |
| Crinosterol | 18 | 0.000199626 | SLC6A2 | P23975 |
| Crinosterol | 19 | 0.000188048 | ACHE | P22303 |
| Crinosterol | 11 | 0.000215313 | CYP19A1 | P11511 |
| Crinosterol | 6 | 0.000279495 | VDR | P11473 |
| Crinosterol | 4 | 0.000300136 | GPER1 | P10275 |
| Crinosterol | 15 | 0.000201888 | NR3C2 | P08235 |
| Crinosterol | 9 | 0.00023801 | PGR | P06401 |
| Crinosterol | 1 | 0.000678622 | SHBG | P04278 |
| Crinosterol | 2 | 0.000579596 | NR3C1 | P04150 |
| Crinosterol | 5 | 0.000283198 | HMGCR | P04035 |
| Crinosterol | 8 | 0.000269704 | ESR1 | P03372 |
| Crinosterol | 10 | 0.000228977 | RBP4 | P02753 |
| Crinosterol | 12 | 0.000214701 | GGPS1 | O95749 |
| Cycloartenol | 14 | 0.000189242 | ESR2 | Q92731 |
| Cycloartenol | 7 | 0.000276285 | GPBAR1 | Q8TDU6 |
| Cycloartenol | 20 | 0.000170158 | SLC10A2 | Q12908 |
| Cycloartenol | 10 | 0.000207585 | RORC | P51449 |
| Cycloartenol | 19 | 0.000172378 | OPRD1 | P41143 |
| Cycloartenol | 3 | 0.000427166 | FDFT1 | P37268 |
| Cycloartenol | 9 | 0.000215365 | OPRM1 | P35372 |
| Cycloartenol | 15 | 0.000185027 | SLC6A4 | P31645 |
| Cycloartenol | 13 | 0.00019301 | SLC6A2 | P23975 |
| Cycloartenol | 17 | 0.000180317 | CYP19A1 | P11511 |
| Cycloartenol | 5 | 0.000318825 | VDR | P11473 |
| Cycloartenol | 6 | 0.000282703 | GPER1 | P10275 |
| Cycloartenol | 16 | 0.000183722 | NR3C2 | P08235 |
| Cycloartenol | 11 | 0.000204371 | PGR | P06401 |
| Cycloartenol | 1 | 0.000569126 | SHBG | P04278 |
| Cycloartenol | 2 | 0.000566207 | NR3C1 | P04150 |
| Cycloartenol | 12 | 0.000203206 | HMGCR | P04035 |
| Cycloartenol | 8 | 0.000241801 | ESR1 | P03372 |
| Cycloartenol | 18 | 0.000173864 | RBP4 | P02753 |
| Cycloartenol | 4 | 0.000384498 | GGPS1 | O95749 |
| Cycloleucalenol | 11 | 0.000196857 | ESR2 | Q92731 |
| Cycloleucalenol | 4 | 0.00028827 | GPBAR1 | Q8TDU6 |
| Cycloleucalenol | 16 | 0.00017979 | SLC10A2 | Q12908 |
| Cycloleucalenol | 8 | 0.000211879 | RORC | P51449 |
| Cycloleucalenol | 19 | 0.000170465 | OPRK1 | P41145 |
| Cycloleucalenol | 18 | 0.000173764 | OPRD1 | P41143 |
| Cycloleucalenol | 7 | 0.000225425 | OPRM1 | P35372 |
| Cycloleucalenol | 13 | 0.000193788 | SLC6A4 | P31645 |
| Cycloleucalenol | 10 | 0.000205197 | SLC6A2 | P23975 |
| Cycloleucalenol | 20 | 0.000165994 | FDPS | P14324 |
| Cycloleucalenol | 17 | 0.000173992 | CYP19A1 | P11511 |
| Cycloleucalenol | 3 | 0.000332058 | VDR | P11473 |
| Cycloleucalenol | 5 | 0.000281997 | GPER1 | P10275 |
| Cycloleucalenol | 15 | 0.000182162 | NR3C2 | P08235 |
| Cycloleucalenol | 12 | 0.000194363 | PGR | P06401 |
| Cycloleucalenol | 1 | 0.000568685 | SHBG | P04278 |
| Cycloleucalenol | 2 | 0.000558751 | NR3C1 | P04150 |
| Cycloleucalenol | 9 | 0.000210595 | HMGCR | P04035 |
| Cycloleucalenol | 6 | 0.000250314 | ESR1 | P03372 |
| Cycloleucalenol | 14 | 0.000188607 | GGPS1 | O95749 |
| Episterol | 17 | 0.000203741 | ESR2 | Q92731 |
| Episterol | 3 | 0.000357074 | GPBAR1 | Q8TDU6 |
| Episterol | 13 | 0.000214053 | SLC10A2 | Q12908 |
| Episterol | 7 | 0.000283496 | RORC | P51449 |
| Episterol | 12 | 0.000217041 | OPRM1 | P35372 |
| Episterol | 16 | 0.000204192 | SLC6A4 | P31645 |
| Episterol | 14 | 0.000212752 | SLC6A2 | P23975 |
| Episterol | 11 | 0.000217388 | CYP19A1 | P11511 |
| Episterol | 4 | 0.000339068 | VDR | P11473 |
| Episterol | 5 | 0.0003094 | GPER1 | P10275 |
| Episterol | 15 | 0.000208194 | NR3C2 | P08235 |
| Episterol | 10 | 0.000256393 | PGR | P06401 |
| Episterol | 1 | 0.000733402 | SHBG | P04278 |
| Episterol | 2 | 0.000601256 | NR3C1 | P04150 |
| Episterol | 8 | 0.000274694 | HMGCR | P04035 |
| Episterol | 9 | 0.000269426 | ESR1 | P03372 |
| Episterol | 6 | 0.0003057 | RBP4 | P02753 |
| Episterol | 18 | 0.00019179 | GGPS1 | O95749 |
| Ethyl acetate | 10 | 0.000188323 | SLC22A11 | Q9NSA0 |
| Ethyl acetate | 16 | 0.000164135 | SLC22A6 | Q4U2R8 |
| Ethyl acetate | 18 | 0.000159737 | KCNH2 | Q12809 |
| Ethyl acetate | 12 | 0.000176716 | PPARA | Q07869 |
| Ethyl acetate | 7 | 0.000220564 | CASP9 | P55211 |
| Ethyl acetate | 15 | 0.000164906 | PPARG | P37231 |
| Ethyl acetate | 5 | 0.000227037 | PTGS2 | P35354 |
| Ethyl acetate | 13 | 0.000176297 | NOS2 | P35228 |
| Ethyl acetate | 14 | 0.000165654 | AGTR1 | P30556 |
| Ethyl acetate | 19 | 0.000159315 | DNMT1 | P26358 |
| Ethyl acetate | 6 | 0.000224337 | PTGS1 | P23219 |
| Ethyl acetate | 8 | 0.000189973 | CYP11B2 | P19099 |
| Ethyl acetate | 20 | 0.000158225 | PTPN1 | P18031 |
| Ethyl acetate | 9 | 0.000189973 | CYP11B1 | P15538 |
| Ethyl acetate | 4 | 0.00023142 | MIF | P14174 |
| Ethyl acetate | 2 | 0.000250178 | ACE | P12821 |
| Ethyl acetate | 17 | 0.000161552 | CYP2C9 | P11712 |
| Ethyl acetate | 11 | 0.000181379 | CHRM1 | P11229 |
| Ethyl acetate | 3 | 0.000232043 | NR3C1 | P04150 |
| Ethyl acetate | 1 | 0.000280504 | GALR3 | O60755 |
| Ethylbenzene | 1 | 0.000607147 | TAAR1 | Q96RJ0 |
| Ethylbenzene | 20 | 0.000186108 | KCNH2 | Q12809 |
| Ethylbenzene | 10 | 0.000228528 | PPARA | Q07869 |
| Ethylbenzene | 14 | 0.000209647 | SLC6A3 | Q01959 |
| Ethylbenzene | 15 | 0.000205053 | PPARG | P37231 |
| Ethylbenzene | 19 | 0.000188672 | PTGS2 | P35354 |
| Ethylbenzene | 13 | 0.000210006 | SLC6A4 | P31645 |
| Ethylbenzene | 12 | 0.000219924 | AGTR1 | P30556 |
| Ethylbenzene | 16 | 0.000202782 | DPP4 | P27487 |
| Ethylbenzene | 18 | 0.000198 | CDK2 | P24941 |
| Ethylbenzene | 8 | 0.000235585 | SLC6A2 | P23975 |
| Ethylbenzene | 9 | 0.000233467 | PTGS1 | P23219 |
| Ethylbenzene | 5 | 0.00025562 | ACHE | P22303 |
| Ethylbenzene | 11 | 0.000220433 | ACPP | P15309 |
| Ethylbenzene | 6 | 0.000254939 | CYP2A6 | P11509 |
| Ethylbenzene | 7 | 0.000242385 | LTA4H | P09960 |
| Ethylbenzene | 17 | 0.000198634 | HSP90AB1 | P08238 |
| Ethylbenzene | 3 | 0.000322763 | ADA | P00813 |
| Ethylbenzene | 4 | 0.000283367 | DHFR | P00374 |
| Ethylbenzene | 2 | 0.000393515 | GGPS1 | O95749 |
| Fernenol | 13 | 0.00020863 | ESR2 | Q92731 |
| Fernenol | 3 | 0.000317862 | GPBAR1 | Q8TDU6 |
| Fernenol | 17 | 0.000189171 | SLC10A2 | Q12908 |
| Fernenol | 9 | 0.000255492 | RORC | P51449 |
| Fernenol | 19 | 0.000176668 | OPRK1 | P41145 |
| Fernenol | 11 | 0.000217534 | OPRM1 | P35372 |
| Fernenol | 16 | 0.000193595 | SLC6A4 | P31645 |
| Fernenol | 15 | 0.000196762 | SLC6A2 | P23975 |
| Fernenol | 20 | 0.000175505 | ACHE | P22303 |
| Fernenol | 12 | 0.00021668 | CYP19A1 | P11511 |
| Fernenol | 6 | 0.000310534 | VDR | P11473 |
| Fernenol | 5 | 0.000313579 | GPER1 | P10275 |
| Fernenol | 14 | 0.000207705 | NR3C2 | P08235 |
| Fernenol | 8 | 0.000259656 | PGR | P06401 |
| Fernenol | 1 | 0.000722894 | SHBG | P04278 |
| Fernenol | 2 | 0.000600991 | NR3C1 | P04150 |
| Fernenol | 10 | 0.000228922 | HMGCR | P04035 |
| Fernenol | 7 | 0.000274629 | ESR1 | P03372 |
| Fernenol | 4 | 0.000316736 | RBP4 | P02753 |
| Fernenol | 18 | 0.000188238 | GGPS1 | O95749 |
| Fungisterol | 17 | 0.000203741 | ESR2 | Q92731 |
| Fungisterol | 3 | 0.000357074 | GPBAR1 | Q8TDU6 |
| Fungisterol | 13 | 0.000214053 | SLC10A2 | Q12908 |
| Fungisterol | 7 | 0.000283496 | RORC | P51449 |
| Fungisterol | 12 | 0.000217041 | OPRM1 | P35372 |
| Fungisterol | 16 | 0.000204192 | SLC6A4 | P31645 |
| Fungisterol | 14 | 0.000212752 | SLC6A2 | P23975 |
| Fungisterol | 11 | 0.000217388 | CYP19A1 | P11511 |
| Fungisterol | 4 | 0.000339068 | VDR | P11473 |
| Fungisterol | 5 | 0.0003094 | GPER1 | P10275 |
| Fungisterol | 15 | 0.000208194 | NR3C2 | P08235 |
| Fungisterol | 10 | 0.000256393 | PGR | P06401 |
| Fungisterol | 1 | 0.000733402 | SHBG | P04278 |
| Fungisterol | 2 | 0.000601256 | NR3C1 | P04150 |
| Fungisterol | 8 | 0.000274694 | HMGCR | P04035 |
| Fungisterol | 9 | 0.000269426 | ESR1 | P03372 |
| Fungisterol | 6 | 0.0003057 | RBP4 | P02753 |
| Fungisterol | 18 | 0.00019179 | GGPS1 | O95749 |
| Gramisterol | 14 | 0.000212378 | ESR2 | Q92731 |
| Gramisterol | 3 | 0.000332196 | GPBAR1 | Q8TDU6 |
| Gramisterol | 17 | 0.000200394 | SLC10A2 | Q12908 |
| Gramisterol | 8 | 0.000266971 | RORC | P51449 |
| Gramisterol | 11 | 0.000225373 | OPRM1 | P35372 |
| Gramisterol | 16 | 0.000204615 | SLC6A4 | P31645 |
| Gramisterol | 15 | 0.000210543 | SLC6A2 | P23975 |
| Gramisterol | 19 | 0.000180242 | ACHE | P22303 |
| Gramisterol | 12 | 0.000218137 | CYP19A1 | P11511 |
| Gramisterol | 5 | 0.000315907 | VDR | P11473 |
| Gramisterol | 4 | 0.000318312 | GPER1 | P10275 |
| Gramisterol | 13 | 0.000213477 | NR3C2 | P08235 |
| Gramisterol | 9 | 0.000260266 | PGR | P06401 |
| Gramisterol | 1 | 0.000738785 | SHBG | P04278 |
| Gramisterol | 2 | 0.000617352 | NR3C1 | P04150 |
| Gramisterol | 10 | 0.0002373 | HMGCR | P04035 |
| Gramisterol | 7 | 0.000279632 | ESR1 | P03372 |
| Gramisterol | 6 | 0.0003057 | RBP4 | P02753 |
| Gramisterol | 18 | 0.00019179 | GGPS1 | O95749 |
| Isobutyric acid | 4 | 0.00042969 | SLC22A6 | Q4U2R8 |
| Isobutyric acid | 6 | 0.000386087 | PDE4D | Q08499 |
| Isobutyric acid | 3 | 0.000458335 | PDE4C | Q08493 |
| Isobutyric acid | 10 | 0.000261475 | PPARA | Q07869 |
| Isobutyric acid | 7 | 0.000370316 | PDE4B | Q07343 |
| Isobutyric acid | 12 | 0.00020353 | RELA | Q04206 |
| Isobutyric acid | 17 | 0.00016823 | PPARD | Q03181 |
| Isobutyric acid | 8 | 0.000283697 | AKR1C3 | P42330 |
| Isobutyric acid | 11 | 0.000238929 | PPARG | P37231 |
| Isobutyric acid | 1 | 0.000671067 | PTGS2 | P35354 |
| Isobutyric acid | 5 | 0.00041908 | PDE4A | P27815 |
| Isobutyric acid | 19 | 0.000160657 | CDK2 | P24941 |
| Isobutyric acid | 2 | 0.000654883 | PTGS1 | P23219 |
| Isobutyric acid | 20 | 0.000159435 | PTPN1 | P18031 |
| Isobutyric acid | 14 | 0.000175974 | CPA1 | P15085 |
| Isobutyric acid | 15 | 0.000170035 | CYP2C9 | P11712 |
| Isobutyric acid | 9 | 0.000269136 | GPER1 | P10275 |
| Isobutyric acid | 16 | 0.000168344 | SHBG | P04278 |
| Isobutyric acid | 18 | 0.00016727 | NR3C1 | P04150 |
| Isobutyric acid | 13 | 0.00019983 | HMGCR | P04035 |
| Isoliquiritigenin | 2 | 0.000802342 | ESR2 | Q92731 |
| Isoliquiritigenin | 10 | 0.000304625 | CYP1B1 | Q16678 |
| Isoliquiritigenin | 16 | 0.000226196 | MAPK14 | Q16539 |
| Isoliquiritigenin | 4 | 0.00047747 | TAS2R31 | P59538 |
| Isoliquiritigenin | 19 | 0.000199405 | PPARG | P37231 |
| Isoliquiritigenin | 14 | 0.00025293 | ACHE | P22303 |
| Isoliquiritigenin | 12 | 0.000268106 | CBR1 | P16152 |
| Isoliquiritigenin | 3 | 0.000662414 | AKR1B1 | P15121 |
| Isoliquiritigenin | 5 | 0.000385898 | CYP19A1 | P11511 |
| Isoliquiritigenin | 6 | 0.000368771 | PPARD | P11309 |
| Isoliquiritigenin | 9 | 0.000313615 | HSP90AB1 | P08238 |
| Isoliquiritigenin | 8 | 0.000318351 | HSP90AA1 | P07900 |
| Isoliquiritigenin | 20 | 0.000197543 | ADRB2 | P07550 |
| Isoliquiritigenin | 15 | 0.000235437 | CYP1A1 | P04798 |
| Isoliquiritigenin | 17 | 0.000223083 | NR3C1 | P04150 |
| Isoliquiritigenin | 1 | 0.001081115 | ESR1 | P03372 |
| Isoliquiritigenin | 11 | 0.000294139 | CA2 | P00918 |
| Isoliquiritigenin | 7 | 0.000329573 | CA1 | P00915 |
| Isoliquiritigenin | 13 | 0.000265732 | CA12 | O43570 |
| Isorhamnetin | 7 | 0.000348599 | ESR2 | Q92731 |
| Isorhamnetin | 9 | 0.00033003 | CYP1B1 | Q16678 |
| Isorhamnetin | 15 | 0.00026061 | MAPK14 | Q16539 |
| Isorhamnetin | 16 | 0.000258714 | MC1R | Q00534 |
| Isorhamnetin | 1 | 0.00049699 | TAS2R31 | P59538 |
| Isorhamnetin | 12 | 0.00028259 | XDH | P47989 |
| Isorhamnetin | 11 | 0.000289491 | HSD17B3 | P37058 |
| Isorhamnetin | 13 | 0.000265958 | ACHE | P22303 |
| Isorhamnetin | 5 | 0.000358537 | ALOX12 | P18054 |
| Isorhamnetin | 6 | 0.000356599 | ALOX15 | P16050 |
| Isorhamnetin | 4 | 0.00039859 | AKR1B1 | P15121 |
| Isorhamnetin | 8 | 0.00033333 | CYP19A1 | P11511 |
| Isorhamnetin | 3 | 0.000444288 | PPARD | P11309 |
| Isorhamnetin | 18 | 0.000236293 | HSP90AB1 | P08238 |
| Isorhamnetin | 17 | 0.000243317 | ADRB2 | P07550 |
| Isorhamnetin | 14 | 0.000263679 | CYP1A1 | P04798 |
| Isorhamnetin | 2 | 0.000460651 | ESR1 | P03372 |
| Isorhamnetin | 19 | 0.000223113 | CA2 | P00918 |
| Isorhamnetin | 20 | 0.000220853 | CA1 | P00915 |
| Isorhamnetin | 10 | 0.000290129 | DHFR | P00374 |
| Kaempferide | 8 | 0.000323723 | ESR2 | Q92731 |
| Kaempferide | 6 | 0.000338245 | CYP1B1 | Q16678 |
| Kaempferide | 12 | 0.000285304 | MAPK14 | Q16539 |
| Kaempferide | 18 | 0.000256255 | PPARA | Q07869 |
| Kaempferide | 14 | 0.000269731 | MC1R | Q00534 |
| Kaempferide | 1 | 0.000539878 | TAS2R31 | P59538 |
| Kaempferide | 11 | 0.000293517 | XDH | P47989 |
| Kaempferide | 17 | 0.000257238 | PPARG | P37231 |
| Kaempferide | 10 | 0.000302049 | HSD17B3 | P37058 |
| Kaempferide | 19 | 0.000254161 | ACHE | P22303 |
| Kaempferide | 7 | 0.00033357 | ALOX12 | P18054 |
| Kaempferide | 5 | 0.000369628 | ALOX15 | P16050 |
| Kaempferide | 4 | 0.000424914 | AKR1B1 | P15121 |
| Kaempferide | 9 | 0.00031887 | CYP19A1 | P11511 |
| Kaempferide | 2 | 0.000454228 | PPARD | P11309 |
| Kaempferide | 13 | 0.000276565 | HSP90AB1 | P08238 |
| Kaempferide | 15 | 0.000265063 | CYP1A1 | P04798 |
| Kaempferide | 3 | 0.000449745 | ESR1 | P03372 |
| Kaempferide | 16 | 0.000258059 | CA2 | P00918 |
| Kaempferide | 20 | 0.000238267 | CA1 | P00915 |
| Liquiritigenin | 3 | 0.000554789 | ESR2 | Q92731 |
| Liquiritigenin | 5 | 0.000296251 | CYP1B1 | Q16678 |
| Liquiritigenin | 19 | 0.000181285 | MAPK14 | Q16539 |
| Liquiritigenin | 6 | 0.000282401 | PPARA | Q07869 |
| Liquiritigenin | 11 | 0.000217501 | PPARD | Q03181 |
| Liquiritigenin | 2 | 0.00061719 | TAS2R31 | P59538 |
| Liquiritigenin | 8 | 0.000274131 | PPARG | P37231 |
| Liquiritigenin | 18 | 0.000181491 | SLC6A2 | P23975 |
| Liquiritigenin | 14 | 0.000190817 | ACHE | P22303 |
| Liquiritigenin | 10 | 0.000237924 | CBR1 | P16152 |
| Liquiritigenin | 7 | 0.000280392 | AKR1B1 | P15121 |
| Liquiritigenin | 17 | 0.000182702 | HSD17B1 | P14061 |
| Liquiritigenin | 4 | 0.000373871 | CYP19A1 | P11511 |
| Liquiritigenin | 20 | 0.000179402 | ADRB1 | P08588 |
| Liquiritigenin | 9 | 0.000241975 | ADRB2 | P07550 |
| Liquiritigenin | 13 | 0.000196523 | CYP1A1 | P04798 |
| Liquiritigenin | 16 | 0.000184017 | SHBG | P04278 |
| Liquiritigenin | 1 | 0.000648314 | ESR1 | P03372 |
| Liquiritigenin | 15 | 0.000187903 | CA2 | P00918 |
| Liquiritigenin | 12 | 0.000214606 | GALR3 | O60755 |
| Lupeol | 10 | 0.000220895 | ESR2 | Q92731 |
| Lupeol | 3 | 0.00033674 | GPBAR1 | Q8TDU6 |
| Lupeol | 12 | 0.000204855 | SLC10A2 | Q12908 |
| Lupeol | 7 | 0.000234719 | RORC | P51449 |
| Lupeol | 17 | 0.000190012 | OPRK1 | P41145 |
| Lupeol | 19 | 0.000181228 | OPRD1 | P41143 |
| Lupeol | 9 | 0.000226478 | OPRM1 | P35372 |
| Lupeol | 15 | 0.000200774 | SLC6A4 | P31645 |
| Lupeol | 18 | 0.0001883 | SLC6A2 | P23975 |
| Lupeol | 20 | 0.000179544 | FDPS | P14324 |
| Lupeol | 14 | 0.000200829 | CYP19A1 | P11511 |
| Lupeol | 6 | 0.000252091 | VDR | P11473 |
| Lupeol | 4 | 0.000301377 | GPER1 | P10275 |
| Lupeol | 16 | 0.000198784 | NR3C2 | P08235 |
| Lupeol | 8 | 0.00023088 | PGR | P06401 |
| Lupeol | 1 | 0.000668671 | SHBG | P04278 |
| Lupeol | 2 | 0.000595475 | NR3C1 | P04150 |
| Lupeol | 11 | 0.000211576 | HMGCR | P04035 |
| Lupeol | 5 | 0.00028821 | ESR1 | P03372 |
| Lupeol | 13 | 0.000204003 | GGPS1 | O95749 |
| Obtusifoliol | 14 | 0.000212378 | ESR2 | Q92731 |
| Obtusifoliol | 3 | 0.000332196 | GPBAR1 | Q8TDU6 |
| Obtusifoliol | 17 | 0.000200394 | SLC10A2 | Q12908 |
| Obtusifoliol | 8 | 0.000266971 | RORC | P51449 |
| Obtusifoliol | 11 | 0.000225373 | OPRM1 | P35372 |
| Obtusifoliol | 16 | 0.000204615 | SLC6A4 | P31645 |
| Obtusifoliol | 15 | 0.000210543 | SLC6A2 | P23975 |
| Obtusifoliol | 19 | 0.000180242 | ACHE | P22303 |
| Obtusifoliol | 12 | 0.000218137 | CYP19A1 | P11511 |
| Obtusifoliol | 5 | 0.000315907 | VDR | P11473 |
| Obtusifoliol | 4 | 0.000318312 | GPER1 | P10275 |
| Obtusifoliol | 13 | 0.000213477 | NR3C2 | P08235 |
| Obtusifoliol | 9 | 0.000260266 | PGR | P06401 |
| Obtusifoliol | 1 | 0.000738785 | SHBG | P04278 |
| Obtusifoliol | 2 | 0.000617352 | NR3C1 | P04150 |
| Obtusifoliol | 10 | 0.0002373 | HMGCR | P04035 |
| Obtusifoliol | 7 | 0.000279632 | ESR1 | P03372 |
| Obtusifoliol | 6 | 0.0003057 | RBP4 | P02753 |
| Obtusifoliol | 18 | 0.00019179 | GGPS1 | O95749 |
| Schottenol | 18 | 0.000200621 | ESR2 | Q92731 |
| Schottenol | 3 | 0.000350461 | GPBAR1 | Q8TDU6 |
| Schottenol | 14 | 0.000210089 | SLC10A2 | Q12908 |
| Schottenol | 7 | 0.000278246 | RORC | P51449 |
| Schottenol | 11 | 0.000216966 | OPRM1 | P35372 |
| Schottenol | 17 | 0.000201893 | SLC6A4 | P31645 |
| Schottenol | 13 | 0.000211435 | SLC6A2 | P23975 |
| Schottenol | 12 | 0.000214113 | CYP19A1 | P11511 |
| Schottenol | 4 | 0.000332789 | VDR | P11473 |
| Schottenol | 5 | 0.000304032 | GPER1 | P10275 |
| Schottenol | 15 | 0.000204339 | NR3C2 | P08235 |
| Schottenol | 10 | 0.000251645 | PGR | P06401 |
| Schottenol | 1 | 0.000722077 | SHBG | P04278 |
| Schottenol | 2 | 0.000590996 | NR3C1 | P04150 |
| Schottenol | 8 | 0.000271833 | HMGCR | P04035 |
| Schottenol | 9 | 0.000266436 | ESR1 | P03372 |
| Schottenol | 6 | 0.000300038 | RBP4 | P02753 |
| Schottenol | 16 | 0.000202093 | GGPS1 | O95749 |
| Sitosterol | 15 | 0.000205277 | ESR2 | Q92731 |
| Sitosterol | 3 | 0.000345228 | GPBAR1 | Q8TDU6 |
| Sitosterol | 17 | 0.000203817 | SLC10A2 | Q12908 |
| Sitosterol | 5 | 0.00027793 | RORC | P51449 |
| Sitosterol | 13 | 0.000207722 | OPRM1 | P35372 |
| Sitosterol | 16 | 0.000204635 | SLC6A4 | P31645 |
| Sitosterol | 14 | 0.000207685 | SLC6A2 | P23975 |
| Sitosterol | 19 | 0.000190043 | ACHE | P22303 |
| Sitosterol | 11 | 0.000209234 | CYP19A1 | P11511 |
| Sitosterol | 6 | 0.000270926 | VDR | P11473 |
| Sitosterol | 4 | 0.000293474 | GPER1 | P10275 |
| Sitosterol | 18 | 0.000197186 | NR3C2 | P08235 |
| Sitosterol | 9 | 0.000233081 | PGR | P06401 |
| Sitosterol | 1 | 0.000670043 | SHBG | P04278 |
| Sitosterol | 2 | 0.000562989 | NR3C1 | P04150 |
| Sitosterol | 7 | 0.000269838 | HMGCR | P04035 |
| Sitosterol | 8 | 0.00026744 | ESR1 | P03372 |
| Sitosterol | 12 | 0.000207976 | RBP4 | P02753 |
| Sitosterol | 10 | 0.000213981 | GGPS1 | O95749 |
| Spinasterol | 17 | 0.000200206 | ESR2 | Q92731 |
| Spinasterol | 3 | 0.000342279 | GPBAR1 | Q8TDU6 |
| Spinasterol | 16 | 0.000202577 | SLC10A2 | Q12908 |
| Spinasterol | 8 | 0.000269802 | RORC | P51449 |
| Spinasterol | 12 | 0.000214764 | OPRM1 | P35372 |
| Spinasterol | 18 | 0.000195316 | SLC6A4 | P31645 |
| Spinasterol | 15 | 0.00020275 | SLC6A2 | P23975 |
| Spinasterol | 19 | 0.000183899 | ACHE | P22303 |
| Spinasterol | 11 | 0.000216619 | CYP19A1 | P11511 |
| Spinasterol | 4 | 0.000335706 | VDR | P11473 |
| Spinasterol | 6 | 0.000305128 | GPER1 | P10275 |
| Spinasterol | 14 | 0.000205041 | NR3C2 | P08235 |
| Spinasterol | 10 | 0.000251894 | PGR | P06401 |
| Spinasterol | 1 | 0.000719868 | SHBG | P04278 |
| Spinasterol | 2 | 0.000596821 | NR3C1 | P04150 |
| Spinasterol | 7 | 0.000281433 | HMGCR | P04035 |
| Spinasterol | 9 | 0.00026558 | ESR1 | P03372 |
| Spinasterol | 5 | 0.000315632 | RBP4 | P02753 |
| Spinasterol | 13 | 0.000212651 | GGPS1 | O95749 |
| Stigmastanol | 15 | 0.000205597 | ESR2 | Q92731 |
| Stigmastanol | 3 | 0.000374922 | GPBAR1 | Q8TDU6 |
| Stigmastanol | 9 | 0.000231527 | SLC10A2 | Q12908 |
| Stigmastanol | 7 | 0.000266048 | RORC | P51449 |
| Stigmastanol | 20 | 0.000177831 | OPRK1 | P41145 |
| Stigmastanol | 10 | 0.000224051 | OPRM1 | P35372 |
| Stigmastanol | 13 | 0.000212761 | SLC6A4 | P31645 |
| Stigmastanol | 12 | 0.000214131 | SLC6A2 | P23975 |
| Stigmastanol | 17 | 0.00018938 | FDPS | P14324 |
| Stigmastanol | 18 | 0.000185214 | CYP19A1 | P11511 |
| Stigmastanol | 5 | 0.000274 | VDR | P11473 |
| Stigmastanol | 4 | 0.000281537 | GPER1 | P10275 |
| Stigmastanol | 16 | 0.000190799 | NR3C2 | P08235 |
| Stigmastanol | 11 | 0.000216098 | PGR | P06401 |
| Stigmastanol | 1 | 0.0006521 | SHBG | P04278 |
| Stigmastanol | 2 | 0.000571389 | NR3C1 | P04150 |
| Stigmastanol | 8 | 0.000262179 | HMGCR | P04035 |
| Stigmastanol | 6 | 0.000271352 | ESR1 | P03372 |
| Stigmastanol | 19 | 0.000180957 | ADA | P00813 |
| Stigmastanol | 14 | 0.000210521 | GGPS1 | O95749 |
| Stigmasterol | 14 | 0.000204838 | ESR2 | Q92731 |
| Stigmasterol | 3 | 0.000336565 | GPBAR1 | Q8TDU6 |
| Stigmasterol | 18 | 0.000195863 | SLC10A2 | Q12908 |
| Stigmasterol | 7 | 0.00026899 | RORC | P51449 |
| Stigmasterol | 13 | 0.00020539 | OPRM1 | P35372 |
| Stigmasterol | 17 | 0.000197672 | SLC6A4 | P31645 |
| Stigmasterol | 15 | 0.000198489 | SLC6A2 | P23975 |
| Stigmasterol | 19 | 0.000191843 | ACHE | P22303 |
| Stigmasterol | 20 | 0.000181953 | FDPS | P14324 |
| Stigmasterol | 12 | 0.000211887 | CYP19A1 | P11511 |
| Stigmasterol | 6 | 0.000274014 | VDR | P11473 |
| Stigmasterol | 4 | 0.000294635 | GPER1 | P10275 |
| Stigmasterol | 16 | 0.000197929 | NR3C2 | P08235 |
| Stigmasterol | 9 | 0.000233344 | PGR | P06401 |
| Stigmasterol | 1 | 0.000667704 | SHBG | P04278 |
| Stigmasterol | 2 | 0.000569157 | NR3C1 | P04150 |
| Stigmasterol | 5 | 0.000280002 | HMGCR | P04035 |
| Stigmasterol | 8 | 0.000266533 | ESR1 | P03372 |
| Stigmasterol | 11 | 0.000224487 | RBP4 | P02753 |
| Stigmasterol | 10 | 0.00022516 | GGPS1 | O95749 |
| Taraxerol | 14 | 0.000204256 | ESR2 | Q92731 |
| Taraxerol | 4 | 0.000320299 | GPBAR1 | Q8TDU6 |
| Taraxerol | 18 | 0.000189508 | SLC10A2 | Q12908 |
| Taraxerol | 9 | 0.000251229 | RORC | P51449 |
| Taraxerol | 19 | 0.000185418 | OPRK1 | P41145 |
| Taraxerol | 11 | 0.000225414 | OPRM1 | P35372 |
| Taraxerol | 16 | 0.000193938 | SLC6A4 | P31645 |
| Taraxerol | 17 | 0.000190879 | SLC6A2 | P23975 |
| Taraxerol | 20 | 0.000180679 | ACHE | P22303 |
| Taraxerol | 12 | 0.000220718 | CYP19A1 | P11511 |
| Taraxerol | 6 | 0.000313429 | VDR | P11473 |
| Taraxerol | 5 | 0.000318575 | GPER1 | P10275 |
| Taraxerol | 13 | 0.00020981 | NR3C2 | P08235 |
| Taraxerol | 8 | 0.000264714 | PGR | P06401 |
| Taraxerol | 1 | 0.00073622 | SHBG | P04278 |
| Taraxerol | 2 | 0.000608513 | NR3C1 | P04150 |
| Taraxerol | 10 | 0.000228501 | HMGCR | P04035 |
| Taraxerol | 7 | 0.000271838 | ESR1 | P03372 |
| Taraxerol | 3 | 0.000342075 | RBP4 | P02753 |
| Taraxerol | 15 | 0.000202362 | GGPS1 | O95749 |
| Terbenthene | 15 | 0.000210105 | GPBAR1 | Q8TDU6 |
| Terbenthene | 18 | 0.000186286 | MAPK14 | Q16539 |
| Terbenthene | 11 | 0.000223559 | OPRK1 | P41145 |
| Terbenthene | 9 | 0.000226869 | OPRD1 | P41143 |
| Terbenthene | 5 | 0.000268757 | OPRM1 | P35372 |
| Terbenthene | 12 | 0.000219858 | SLC6A4 | P31645 |
| Terbenthene | 19 | 0.000169324 | CDK2 | P24941 |
| Terbenthene | 10 | 0.000225307 | SLC6A2 | P23975 |
| Terbenthene | 17 | 0.000186592 | ACHE | P22303 |
| Terbenthene | 13 | 0.000219076 | CYP19A1 | P11511 |
| Terbenthene | 4 | 0.000340866 | VDR | P11473 |
| Terbenthene | 7 | 0.000242492 | GPER1 | P10275 |
| Terbenthene | 6 | 0.00024421 | ADH1A | P07327 |
| Terbenthene | 8 | 0.000230831 | PGR | P06401 |
| Terbenthene | 1 | 0.00056916 | SHBG | P04278 |
| Terbenthene | 3 | 0.000436154 | NR3C1 | P04150 |
| Terbenthene | 14 | 0.000212042 | ESR1 | P03372 |
| Terbenthene | 2 | 0.00045436 | RBP4 | P02753 |
| Terbenthene | 16 | 0.000205675 | GGPS1 | O95749 |
| Vernoflexin | 8 | 0.0002048 | SLCO1B1 | Q9Y6L6 |
| Vernoflexin | 12 | 0.000172332 | GPBAR1 | Q8TDU6 |
| Vernoflexin | 17 | 0.000151737 | PPARA | Q07869 |
| Vernoflexin | 19 | 0.000148243 | PRKCE | Q02156 |
| Vernoflexin | 13 | 0.000161322 | NR0B1 | P51843 |
| Vernoflexin | 18 | 0.00015167 | IARS | P41252 |
| Vernoflexin | 16 | 0.000154232 | OPRM1 | P35372 |
| Vernoflexin | 6 | 0.000224314 | GLRA2 | P23416 |
| Vernoflexin | 7 | 0.000210579 | GLRA1 | P23415 |
| Vernoflexin | 15 | 0.0001561 | MIF | P14174 |
| Vernoflexin | 11 | 0.000174835 | CHRM1 | P11229 |
| Vernoflexin | 5 | 0.000229424 | GPER1 | P10275 |
| Vernoflexin | 4 | 0.00023722 | NR3C2 | P08235 |
| Vernoflexin | 10 | 0.000186246 | PGR | P06401 |
| Vernoflexin | 9 | 0.000190248 | PRKCB | P05771 |
| Vernoflexin | 2 | 0.000396639 | SHBG | P04278 |
| Vernoflexin | 1 | 0.000434976 | NR3C1 | P04150 |
| Vernoflexin | 3 | 0.0003648 | HMGCR | P04035 |
| Vernoflexin | 14 | 0.000159128 | ESR1 | P03372 |
| Vernolic acid | 5 | 0.000363442 | OXER1 | Q8TDS5 |
| Vernolic acid | 16 | 0.00020834 | SLC22A6 | Q4U2R8 |
| Vernolic acid | 1 | 0.000475594 | PPARA | Q07869 |
| Vernolic acid | 3 | 0.000399653 | PPARD | Q03181 |
| Vernolic acid | 15 | 0.000223616 | FABP5 | Q01469 |
| Vernolic acid | 13 | 0.000237171 | LSS | P48449 |
| Vernolic acid | 18 | 0.000192263 | IARS | P41252 |
| Vernolic acid | 10 | 0.000247338 | PPARG | P37231 |
| Vernolic acid | 14 | 0.00022956 | PTGS1 | P23219 |
| Vernolic acid | 17 | 0.000201183 | FABP4 | P15090 |
| Vernolic acid | 9 | 0.000248782 | IDE | P14735 |
| Vernolic acid | 7 | 0.000315768 | FDPS | P14324 |
| Vernolic acid | 6 | 0.00036302 | FABP3 | P05413 |
| Vernolic acid | 12 | 0.000244804 | SHBG | P04278 |
| Vernolic acid | 11 | 0.000246072 | NR3C1 | P04150 |
| Vernolic acid | 8 | 0.000261058 | HMGCR | P04035 |
| Vernolic acid | 2 | 0.000469387 | GGPS1 | O95749 |
| Vernolic acid | 4 | 0.000388451 | TLR2 | O60603 |
| Vernolide B | 7 | 0.000175715 | SLCO1B1 | Q9Y6L6 |
| Vernolide B | 19 | 0.000142501 | SLC6A3 | Q01959 |
| Vernolide B | 9 | 0.000166493 | NR0B1 | P51843 |
| Vernolide B | 14 | 0.000146114 | IARS | P41252 |
| Vernolide B | 20 | 0.000141929 | OPRK1 | P41145 |
| Vernolide B | 8 | 0.000168139 | OPRM1 | P35372 |
| Vernolide B | 17 | 0.000142636 | PTGS2 | P35354 |
| Vernolide B | 15 | 0.000145696 | SLC6A2 | P23975 |
| Vernolide B | 11 | 0.000162665 | CYP2C9 | P11712 |
| Vernolide B | 16 | 0.000145198 | TOP1 | P11387 |
| Vernolide B | 10 | 0.000165905 | CHRM1 | P11229 |
| Vernolide B | 4 | 0.000229744 | GPER1 | P10275 |
| Vernolide B | 5 | 0.000189432 | NR3C2 | P08235 |
| Vernolide B | 18 | 0.000142563 | CHRM2 | P08172 |
| Vernolide B | 6 | 0.00018867 | PGR | P06401 |
| Vernolide B | 2 | 0.000347985 | SHBG | P04278 |
| Vernolide B | 1 | 0.00043745 | NR3C1 | P04150 |
| Vernolide B | 3 | 0.000285276 | HMGCR | P04035 |
| Vernolide B | 13 | 0.000152795 | ESR1 | P03372 |
| Vernosterol | 17 | 0.000200712 | ESR2 | Q92731 |
| Vernosterol | 3 | 0.000350461 | GPBAR1 | Q8TDU6 |
| Vernosterol | 14 | 0.000210089 | SLC10A2 | Q12908 |
| Vernosterol | 8 | 0.000278246 | RORC | P51449 |
| Vernosterol | 12 | 0.000215302 | OPRM1 | P35372 |
| Vernosterol | 16 | 0.000203039 | SLC6A4 | P31645 |
| Vernosterol | 13 | 0.000212817 | SLC6A2 | P23975 |
| Vernosterol | 11 | 0.000217674 | CYP19A1 | P11511 |
| Vernosterol | 4 | 0.000339953 | VDR | P11473 |
| Vernosterol | 6 | 0.000306856 | GPER1 | P10275 |
| Vernosterol | 15 | 0.00020686 | NR3C2 | P08235 |
| Vernosterol | 10 | 0.000255457 | PGR | P06401 |
| Vernosterol | 1 | 0.000719821 | SHBG | P04278 |
| Vernosterol | 2 | 0.000605097 | NR3C1 | P04150 |
| Vernosterol | 7 | 0.000285481 | HMGCR | P04035 |
| Vernosterol | 9 | 0.000264676 | ESR1 | P03372 |
| Vernosterol | 5 | 0.000315632 | RBP4 | P02753 |
| Vernosterol | 18 | 0.000198797 | GGPS1 | O95749 |
