# Supplementary material for: Network pharmacological mechanisms of Vernonia anthelmintica (L.) in the treatment of vitiligo: Isorhamnetin induction of melanogenesis via up-regulation of melanin-biosynthetic genes
Source: BMC Syst Biol. 2017 Nov 16;11:103. doi: 10.1186/s12918-017-0486-1 (PMC5691595; doi:10.1186/s12918-017-0486-1)
Supplement: Supplementary file 4 — The information of tissue-specific targets. (DOC 144 kb) (DOC 144 kb) [file 12918_2017_486_MOESM4_ESM.doc]

**Table S4. The information of tissue-specific targets**

| **Target** | **Keratinocytes** | **Melanocytes** |
| --- | --- | --- |
| TAAR1 | Low | No |
| SLC6A4 | Yes (PMID: 19263059) | No |
| PDE4B | Medium | No |
| OPRM1 | Yes (PMID: 27958613) | No |
| GPBAR1 | Low | No |
| GALR3 | Yes (PMID: 16787514) | No |
| FDPS | Low | No |
| FABP5 | Medium | No (PMID: 12459653) |
| ESR1 | Yes (PMID: 27035208) | No (PMID: 27115344) |
| CYP2C9 | Yes (PMID: 12021553) | No |
| CA12 | Medium | No (PMID: 7530513) |
| ALOX15 | Medium | No |
| SHBG | No | Yes (PMID: 12753385) |
| KCNH2 | No | Yes (PMID: 7886006) |
| IARS | No | Medium |
| GLRA2 | No | Low |
| CYP2A6 | No | Yes (PMID: 12021553) |
| XDH | Yes (PMID: 18328088) | Yes (PMID: 18328088) |
| VDR | Yes (PMID: 19815065) | Yes (PMID: 26677265) |
| TOP1 | High | Medium |
| TLR2 | Yes (PMID: 27421917) | Yes (PMID: 21165228) |
| RORA | High | Medium |
| RELA | Medium | Medium |
| PTPN1 | Medium | Low |
| PTGS2 | Low | Low |
| PTGS1 | High | Medium |
| PRKCE | Yes (PMID:28186503) | Low |
| PRKCB | Low | Yes (PMID: 22578192) |
| PPARG | Medium | Yes (PMID: 15030328) |
| PPARD | Medium | Yes (PMID: 15030328) |
| PPARA | Yes (PMID: 19458633) | Yes (PMID: 15030328) |
| PGR | Low | Low |
| PDE4D | Low | Low |
| OXER1 | High | Medium |
| NR3C2 | Medium | Medium |
| NR3C1 | High | Medium |
| NOS2 | Yes (PMID: 10699762) | Yes (PMID:26259928) |
| MIF | Low | Low |
| MC1R | Yes (PMID: 27061711) | Yes (PMID: 28094871) |
| MAPK14 | Low | Yes (PMID: 27287415) |
| LTA4H | Medium | Low |
| LSS | Medium | Low |
| KARS | Medium | Medium |
| IDE | Low | Low |
| HSP90AB1 | Medium | Medium |
| HMGCR | Medium | Low |
| GRIA4 | Yes (PMID: 28210712) | Yes (PMID: 27596138) |
| GRIA2 | Yes (PMID: 28210712) | Yes (PMID: 27596138) |
| GRIA1 | Yes (PMID: 28210712) | Yes (PMID: 27596138) |
| GPER1 | Medium | Medium |
| GLRA1 | Yes (PMID: 25644897) | Yes (PMID: 20959630) |
| DPP4 | Yes (PMID: 26136686) | Yes (PMID: 26829028) |
| DNMT1 | Low | Yes (PMID: 26504497) |
| DHFR | Low | Medium |
| CYP1B1 | Low | Low |
| CYP1A1 | Low | Low |
| CYP11B1 | Yes (PMID: 21239489) | Yes (PMID: 21673307) |
| CHRM2 | Medium | Medium |
| CDK2 | Low | Yes19995375 |
| CBR1 | High | High |
| CASP9 | Low | Low |
| CA2 | Medium | Low |
| ALOX12 | High | High |
| AKR1C3 | Yes (PMID: 19320734) | Yes (PMID: 19320734) |
| AKR1B1 | Low | Low |
| ADRB3 | Yes (PMID: 24760207) | Yes (PMID: 22670614) |
| ADRB2 | Yes (PMID: 17903623) | Yes (PMID: 22670614) |
| ADRB1 | Yes (PMID: 17903623) | Yes (PMID: 22670614) |
| ADA | Yes (PMID:16410722) | Yes (PMID: 8096237) |
| ACHE | Yes (PMID: 21286734) | Yes (PMID: 16962996) |
| ACE | Yes (PMID: 21286734) | Medium |
| ABCC1 | Low | Low |
| TAS2R31 | No | No |
| SLCO1B1 | No | No |
| SLC6A3 | No | No |
| SLC6A2 | No | No |
| SLC22A6 | No | No |
| SLC22A11 | No | No |
| SLC10A2 | No | No |
| RORC | No | No |
| RBP4 | No | No |
| PDE4C | No | No |
| PDE4A | No | No |
| OPRK1 | No | No |
| OPRD1 | No | No |
| NR0B1 | No | No |
| HSP90AA1 | No | No |
| HSD17B3 | No | No |
| HSD17B1 | No | No |
| GGPS1 | No | No |
| FDFT1 | No | No |
| FABP4 | No | No |
| FABP3 | No | No |
| ESR2 | No | No (PMID: 27115344) |
| CYP19A1 | No | No |
| CYP11B2 | No | No |
| CPA1 | No | No |
| CHRM1 | No | No |
| CA1 | No | No |
| AGTR1 | No | No |
| ADH1A | No | No |
| ACPP | No | No |
