# Supplementary material for: Network pharmacological mechanisms of Vernonia anthelmintica (L.) in the treatment of vitiligo: Isorhamnetin induction of melanogenesis via up-regulation of melanin-biosynthetic genes
Source: BMC Syst Biol. 2017 Nov 16;11:103. doi: 10.1186/s12918-017-0486-1 (PMC5691595; doi:10.1186/s12918-017-0486-1)
Supplement: Supplementary file 5 — The pathway analysis of 72 candidate targets via DAVID database. (DOC 51 kb) [file 12918_2017_486_MOESM5_ESM.doc]

**Table S5. The pathway analysis of 72 candidate targets via DAVID database**

| **Category** | **Term** | **PValue** | **Genes** |
| --- | --- | --- | --- |
| KEGG_PATHWAY | hsa04080:Neuroactive ligand-receptor interaction | 1.58E-06 | OPRM1, GLRA1, GLRA2, NR3C1, GRIA4, ADRB3, ADRB2, ADRB1, MC1R, GRIA2, CHRM2, GRIA1, GALR3, TAAR1 |
| KEGG_PATHWAY | hsa00590:Arachidonic acid metabolism | 1.70E-06 | AKR1C3, CBR1, ALOX15, PTGS2, CYP2C9, PTGS1, LTA4H, ALOX12 |
| KEGG_PATHWAY | hsa04024:cAMP signaling pathway | 9.77E-05 | PPARA, ADRB2, ADRB1, GRIA2, GRIA1, CHRM2, RELA, PDE4B, PDE4D, GRIA4 |
| KEGG_PATHWAY | hsa04726:Serotonergic synapse | 6.23E-04 | ALOX15, PTGS2, CYP2C9, SLC6A4, PTGS1, PRKCB, ALOX12 |
| KEGG_PATHWAY | hsa05204:Chemical carcinogenesis | 9.44E-04 | CBR1, CYP1B1, CYP1A1, PTGS2, CYP2C9, CYP2A6 |
| KEGG_PATHWAY | hsa04923:Regulation of lipolysis in adipocytes | 0.001920736 | ADRB3, ADRB2, ADRB1, PTGS2, PTGS1 |
| KEGG_PATHWAY | hsa04723:Retrograde endocannabinoid signaling | 0.002665686 | GRIA2, PTGS2, GRIA1, MAPK14, GRIA4, PRKCB |
| KEGG_PATHWAY | hsa05140:Leishmaniasis | 0.004566259 | PTGS2, MAPK14, RELA, TLR2, NOS2 |
| KEGG_PATHWAY | hsa00980:Metabolism of xenobiotics by cytochrome P450 | 0.005293345 | CBR1, CYP1B1, CYP1A1, CYP2C9, CYP2A6 |
| KEGG_PATHWAY | hsa05222:Small cell lung cancer | 0.008615322 | CASP9, PTGS2, RELA, NOS2, CDK2 |
| KEGG_PATHWAY | hsa04913:Ovarian steroidogenesis | 0.011213697 | AKR1C3, CYP1B1, CYP1A1, PTGS2 |
| KEGG_PATHWAY | hsa05014:Amyotrophic lateral sclerosis (ALS) | 0.011849227 | GRIA2, CASP9, GRIA1, MAPK14 |
| KEGG_PATHWAY | hsa05142:Chagas disease (American trypanosomiasis) | 0.017105049 | ACE, MAPK14, RELA, TLR2, NOS2 |
| KEGG_PATHWAY | hsa00140:Steroid hormone biosynthesis | 0.017675351 | AKR1C3, CYP1B1, CYP1A1, CYP11B1 |
| KEGG_PATHWAY | hsa01100:Metabolic pathways | 0.018276858 | XDH, PTGS2, CYP1A1, CYP11B1, CYP2C9, HMGCR, PTGS1, FDPS, LSS, ADA, AKR1C3, CBR1, ALOX15, DHFR, AKR1B1, DNMT1, CYP2A6, LTA4H, NOS2, ALOX12 |
| KEGG_PATHWAY | hsa04370:VEGF signaling pathway | 0.02020471 | CASP9, PTGS2, MAPK14, PRKCB |
| KEGG_PATHWAY | hsa04924:Renin secretion | 0.022923705 | ADRB3, ACE, ADRB2, ADRB1 |
| KEGG_PATHWAY | hsa05031:Amphetamine addiction | 0.024841887 | GRIA2, GRIA1, GRIA4, PRKCB |
| KEGG_PATHWAY | hsa03320:PPAR signaling pathway | 0.025832627 | PPARA, PPARD, PPARG, FABP5 |
| KEGG_PATHWAY | hsa05145:Toxoplasmosis | 0.025866673 | CASP9, MAPK14, RELA, TLR2, NOS2 |
| KEGG_PATHWAY | hsa05152:Tuberculosis | 0.026484051 | VDR, CASP9, MAPK14, RELA, TLR2, NOS2 |
| KEGG_PATHWAY | hsa04071:Sphingolipid signaling pathway | 0.027302932 | MAPK14, RELA, ABCC1, PRKCE, PRKCB |
| KEGG_PATHWAY | hsa04020:Calcium signaling pathway | 0.027630353 | ADRB3, ADRB2, ADRB1, CHRM2, NOS2, PRKCB |
| KEGG_PATHWAY | hsa05200:Pathways in cancer | 0.032534216 | HSP90AB1, PPARD, CASP9, PTGS2, RELA, PPARG, NOS2, CDK2, PRKCB |
| KEGG_PATHWAY | hsa04728:Dopaminergic synapse | 0.033521659 | GRIA2, GRIA1, MAPK14, GRIA4, PRKCB |
| KEGG_PATHWAY | hsa00232:Caffeine metabolism | 0.046866566 | XDH, CYP2A6 |
| KEGG_PATHWAY | hsa04970:Salivary secretion | 0.048613888 | ADRB3, ADRB2, ADRB1, PRKCB |
| KEGG_PATHWAY | hsa05161:Hepatitis B | 0.049289327 | CASP9, RELA, TLR2, CDK2, PRKCB |
