# Supplementary figures and images for: Network pharmacological mechanisms of Vernonia anthelmintica (L.) in the treatment of vitiligo: Isorhamnetin induction of melanogenesis via up-regulation of melanin-biosynthetic genes
Source: BMC Syst Biol. 2017 Nov 16;11:103. doi: 10.1186/s12918-017-0486-1 (PMC5691595; doi:10.1186/s12918-017-0486-1)

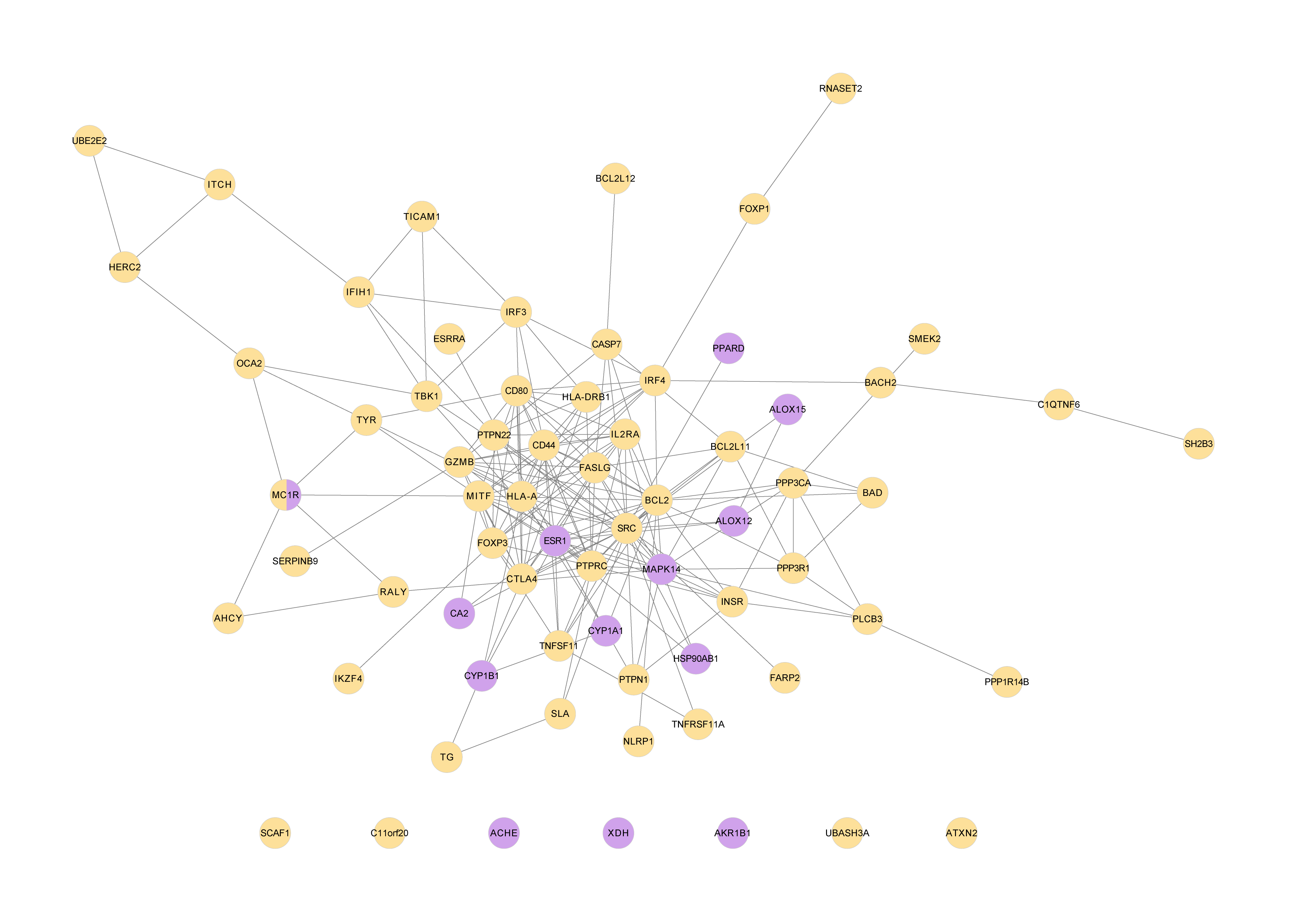

Supplement: Supplementary file 6 — STRING identified a large potential interaction network between 56 vitiligo risk genetic loci and Isorhamnetin/Kaempferide with 13 common targets. The purple nodes represent the targets predicted by SDTNBI. The yellow nodes are closely related to vitiligo risk genetic loci. (TIFF 458 kb) [file 12918_2017_486_MOESM6_ESM.tif]

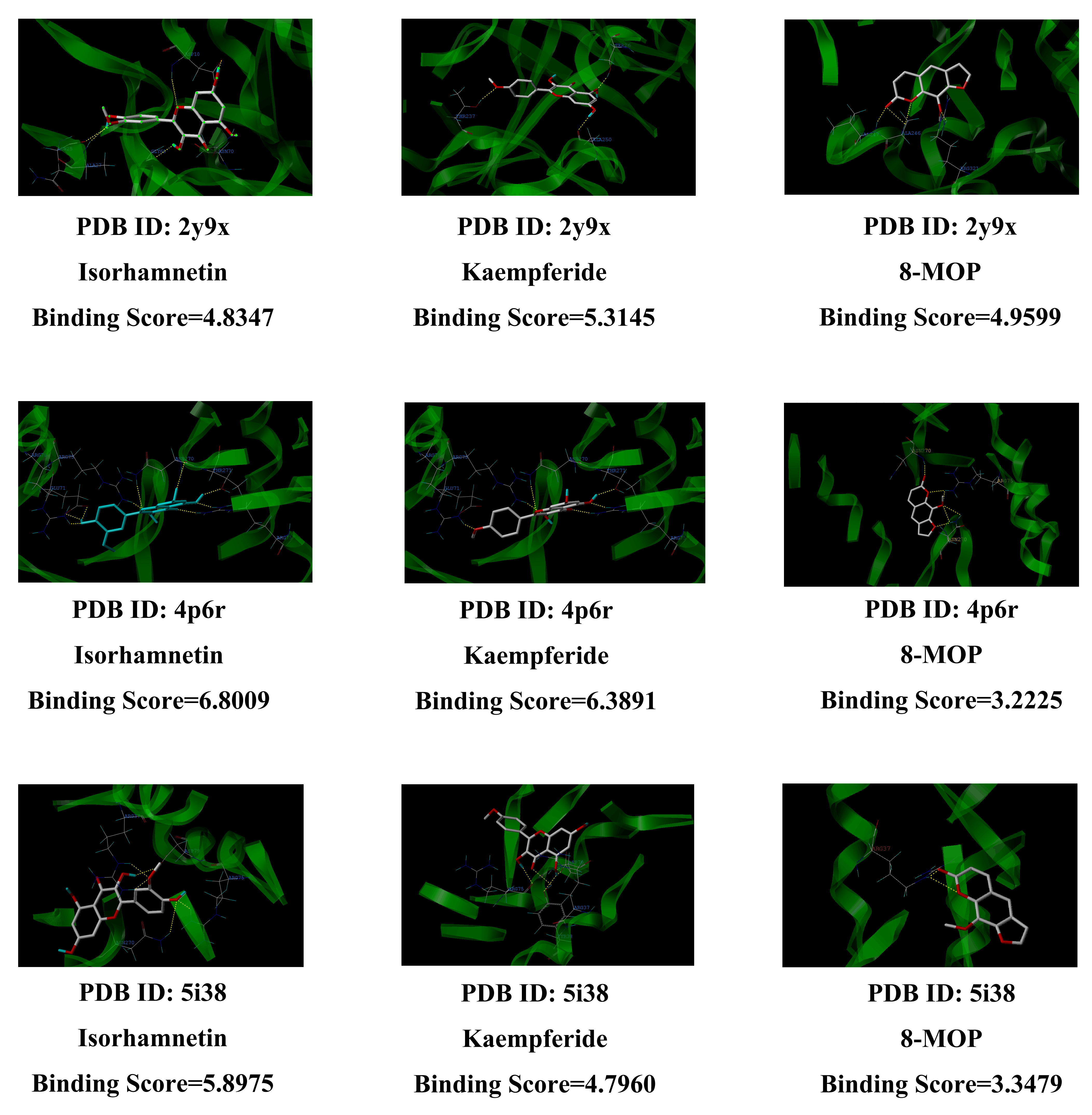

Supplement: Supplementary file 7 — Computational Docking Simulations of Binding between Tyrosinase structure and Isorhamnetin/Kaempferide/8-MOP. The three different crystal structures (PDB ID: 2y9x; 4p6r; 5i38) were used to simulate the 3D tyrosinase structure. The Yellow dotted line represented possible hydrogen-bonding interactions of Isorhamnetin/Kaempferide/8-MOP by the software SYBYL-X 2.0. (TIFF 1550 kb) [file 12918_2017_486_MOESM7_ESM.tif]
